# Highly Selective Adsorption of Para‐Xylene, Ethylbenzene, and Explicit Exclusion of Ortho‐Xylene from Xylene Isomers Using a Pillar‐Layered MOF with Tuned Pore Channels

**DOI:** 10.1002/anie.202512244

**Published:** 2025-07-18

**Authors:** Seonghwan Lee, Amitosh Sharma, Jae Hyeok Lee, Jaewoong Lim, Seung Kyu Min, Hyungphil Chun, Myoung Soo Lah

**Affiliations:** ^1^ Department of Chemistry Ulsan National Institute of Science and Technology (UNIST) Ulsan 44919 Republic of Korea; ^2^ Department of Science Education Ewha Womans University Seoul 03760 Republic of Korea; ^3^ Department of Chemical and Molecular Engineering Hanyang University Ansan 15588 Republic of Korea

**Keywords:** Channel engineering, Metal–organic Framework (MOF), Molecular sieving, Pillar–layered MOF, Xylene Isomer separation

## Abstract

Xylene isomer separation is a long‐standing challenge due to the nearly identical properties of para‐xylene (PX), meta‐xylene (MX), ortho‐xylene (OX), and ethylbenzene (EB). Here, we report a rationally designed pillar‐layered metal–organic framework (MOF), Ni‐HDB, incorporating a cylindrical 1,4‐diazabicyclo[2.2.2]octane (DABCO) pillar that blocks lateral channels and directs molecular transport through elliptical windows (3.2 × 6.7 Å^2^). These apertures closely match the dimensions of PX and EB, enabling kinetic sieving. As a result, Ni‐HDB exhibits high selectivity for PX and EB, moderate selectivity for MX, and exclusion of OX under ambient conditions. It achieves record liquid‐phase selectivities for EB/OX (1943), PX/OX (951), and MX/OX (158), along with high PX and MX adsorption capacities. Comparative studies with isoreticular analogues confirm that DABCO‐driven confinement is key to enhancing size‐based selectivity. Density functional theory calculations indicate kinetic preference for PX and EB, thermodynamic favorability for MX, and exclusion of OX. Ni‐HDB also shows excellent thermal and structural stability, with no performance loss over ten cycles. These results highlight the importance of channel geometry in MOFs and provide a framework for developing next‐generation adsorbents for energy‐efficient hydrocarbon separations.

## Graphical abstract

Channel confinement via rigid cylindrical pillaring in a MOF framework enables molecular sieving through elliptical windows, offering a blueprint for ambient‐condition xylene isomer separation.

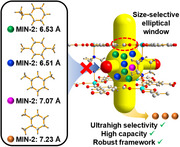

## Introduction

Xylene isomers, including para‐xylene (PX), meta‐xylene (MX), ortho‐xylene (OX), and ethylbenzene (EB), are valuable chemical feedstocks used extensively in the production of polymers, fibers, fuels, and textiles.^[^ ^1^, ^2^ ^]^ Efficient separation of these isomers from reformate mixtures is therefore essential. However, their separation remains one of the most formidable challenges in chemical separations due to their nearly identical molecular sizes, boiling points, and physicochemical properties.^[^ ^3^ ^]^ As a result, xylene isomer separation is listed among the top seven most difficult separations in the chemical industry.^[^ ^3^ ^]^ Current separation methods, such as adsorptive separation for PX/MX/EB and distillation for OX, require harsh operating conditions. For instance, simulated moving bed (SMB) processes demand elevated temperatures (−180 °C) and pressures (−9 bar), while distillation often requires more than 120 theoretical stages.^[^ ^2^, ^4^, ^5^, ^6^ ^]^ These energy‐intensive processes not only lead to substantial greenhouse gas emissions but also pose economic and environmental burdens. This underscores the urgent need for advanced adsorbents that combine high selectivity and adsorption capacity with the ability to operate under energy‐efficient, ambient conditions.^[^ ^3^, ^7^, ^8^, ^9^, ^10^, ^11^, ^12^, ^13^, ^14^, ^15^, ^16^ ^]^

Metal–organic frameworks (MOFs), a class of porous crystalline materials with tunable pore environments, have emerged as promising candidates for such separations.^[^ ^17^, ^18^, ^19^, ^20^, ^21^ ^]^ Their modular nature enables precise tailoring of both kinetic and thermodynamic parameters that govern molecular diffusion and adsorption selectivity.^[^ ^22^, ^23^, ^24^, ^25^, ^26^, ^27^, ^28^, ^29^, ^30^, ^31^, ^32^, ^33^, ^34^, ^35^, ^36^ ^]^ Among various strategies, isoreticular synthesis offers a systematic approach to tuning pore structures by varying metal nodes,^[^ ^37^ ^]^ organic linkers,^[^ ^38^, ^39^, ^40^, ^41^ ^]^ or introducing mixed‐ligand systems.^[^ ^42^, ^43^, ^44^ ^]^ These modifications allow the construction of size‐exclusive diffusion channels and selective interactions critical for xylene isomer discrimination.

Within the broader family of MOFs, pillar‐layered architectures—typically of the form [(Metal)_x_(Linker)_y_(Pillar)_z_]—offer an ideal platform for pore engineering.^[^ ^45^ ^]^ These frameworks consist of two‐periodic metal‐organic layers interconnected by pillaring linkers. Notably, the substitution of different pillaring units does not disrupt the overall topology, thereby enabling systematic modulation of channel accessibility and geometry. One particularly promising system is the pillar‐layered MOF [Ni_3_(H_1.5_BTC)_2_(BTC)(Pillar)_3_], constructed from benzene‐1,3,5‐tricarboxylic acid (H₃BTC) and various pillars including 1,4‐diazabicyclo[2.2.2]octane (DABCO), 4,4′‐bipyridine (BP), and pyrazine (PZ). This framework features elliptical windows (3.2 × 6.7 Å^2^) aligned along the *c*‐axis, which are size‐matched to PX and EB, with minimum dimension‐2 (MIN‐2) values of approximately 6.51 and 6.53 Å, respectively (Table S1). Given the slightly larger molecular sizes of MX (−7.07 Å) and OX (−7.23 Å), these static windows can promote molecular sieving. However, the presence of more accessible diffusion pathways along the *ab*‐plane, particularly in frameworks with either longer or shorter planar pillars, hinders selective separation.

**Scheme 1:**
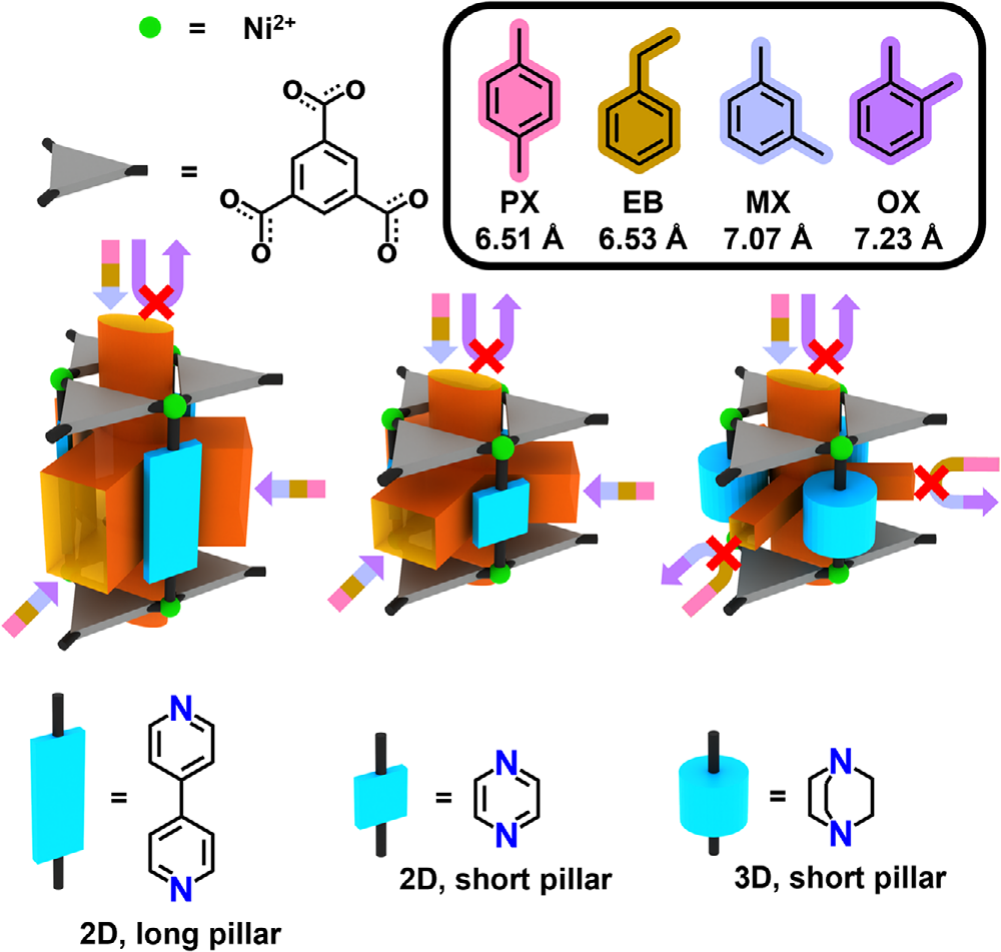
Schematic depiction of this work. Control over channel accessibility is achieved through an isoreticular synthesis approach, wherein the introduction of a shorter cylindrical 3D pillar enables selective use of the small, elliptical window in the one‐periodic channel, whose dimensions closely match those of PX and EB.

To overcome this limitation, we explore the use of DABCO—a short, rigid, cylindrical three‐dimensional (3D) pillar—to restrict diffusion pathways and fully exploit the sieving potential of the elliptical windows in the *c*‐axis. By comparing DABCO‐based Ni‐HDB with its isoreticular analogs Ni‐HBP (BP pillar)^[^ ^46^, ^47^ ^]^ and Ni‐HPZ (PZ pillar),^[^ ^48^ ^]^ we demonstrate that channel control via pillaring geometry offers a powerful strategy for tuning molecular transport and achieving highly selective xylene isomer separation (Scheme 1 and Figure S1).

In Ni‐HBP, the longer, planar two‐dimensional (2D) pillar (BP) produces an open framework with expansive interlayer windows—the largest aperture diameter (LAD) of Window‐AB and Window‐AC is approximately −10.5 Å (Figure S2)—facilitating unimpeded diffusion along the *ab*‐plane and precluding effective sieving. Although Ni‐HPZ incorporates a shorter, planar 2D pillar (PZ), which reduces interlayer spacing and partially narrows the lateral channels, its rotational degrees of freedom result in variable window dimensions—the LAD of Window‐AB and Window‐AC can reach up to 7.9 Å (Figure S3)—which hinder consistent molecular discrimination. In contrast, DABCO introduces a rigid, cylindrical 3D geometry whose dimensions are unaffected by rotational flexibility,^[^ ^49^ ^]^ thereby effectively narrowing lateral apertures and blocking *ab*‐plane diffusion. This enforces transport exclusively along the *c*‐axis through narrow elliptical windows (−3.2 × 6.7 Å^2^), size‐matched to PX and EB. This channel confinement strategy confers three distinct advantages: i) Molecular sieving via a one‐periodic channel through Window‐AA promotes size‐selective exclusion of larger isomers (e.g., OX);^[^ ^49^ ^]^ ii) Nonpolar pore environment, introduced by the aliphatic DABCO linker, enhances C─H⋯C interactions, enabling fine differentiation among xylene isomers based on the orientation of methyl (or ethyl) groups;^[^ ^50^ ^]^ iii) Structural robustness under ambient conditions improves recyclability, simplifies processing, and enables practical deployment.^[^ ^51^ ^]^

In this work, we demonstrate that DABCO‐based Ni‐HDB exhibits record‐high selectivities in the liquid‐phase separation of EB/OX (1943), PX/OX (951), and MX/OX (158), while OX is explicitly excluded from adsorption. High adsorption capacities for PX and MX, operation under ambient‐conditions, and excellent reusability further underscore the framework's potential for energy‐efficient, industrial‐scale xylene separation. Combined structural, adsorption, and computational analyses support a mechanistic model wherein kinetic sieving dominates at ambient temperatures, while thermodynamic preferences emerge at elevated conditions. This study establishes a generalizable design principle for advanced MOF‐based molecular sieves through isoreticular control of diffusion pathways.

## Results and Discussion

The [Ni_3_(H_1.5_BTC)_2_(BTC)(Pillar)_3_] framework consists of neutral, ditopic pillaring linkers that bridge Ni^2^⁺ centers, interconnecting two‐periodic Ni‐BTC layers to generate a three‐periodic network (Figure S1). Various derivatives of this structure have been reported using synthetic strategies such as post‐synthetic ligand exchange, linker insertion, or direct solvothermal assembly.^[^ ^46^, ^47^, ^48^, ^52^ ^]^ All frameworks in this series share a common (3,5)‐c **hms** topology, featuring three types of pores—Pore‐A, Pore‐B, and Pore‐C (Figures S1 and S4).

**Figure 1:**
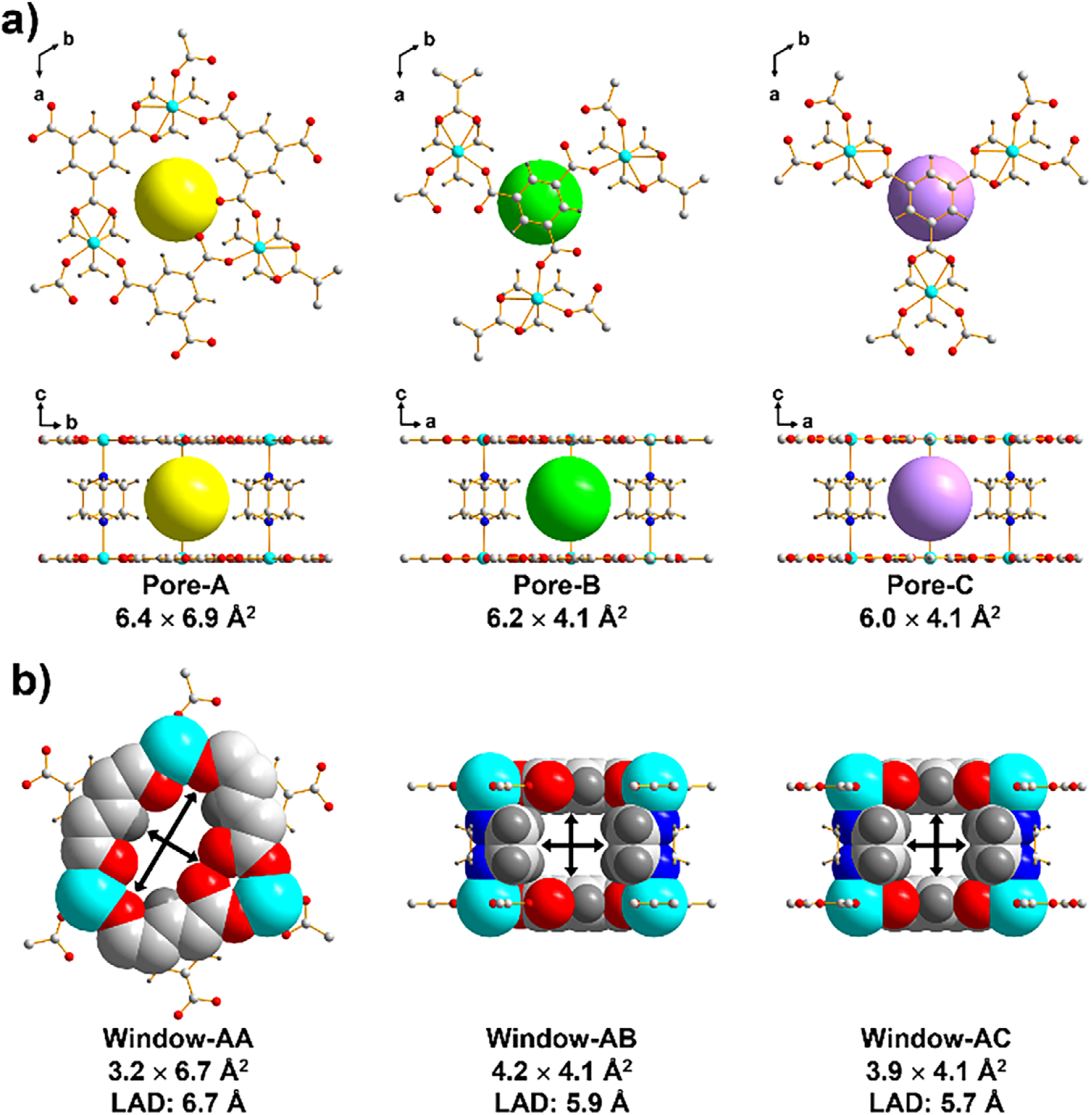
The pores and windows of Ni‐HDB. a) Graphical representations of each pore, Pore‐A, Pore‐B, and Pore‐C, with top view (top) and side view (bottom) provided. b) Graphical representations of each window. LAD is calculated as the largest diagonal distance in the window, considering the possibility of pillar rotation.

Pore‐A, the largest pore, is aligned along the *c*‐axis and connected to adjacent Pore‐A units via a narrow, elliptical window (Window‐AA). The LAD of this window is approximately 6.7 Å, which is sufficiently narrow to enable kinetic discrimination of xylene isomers (Table S1), particularly favoring PX. In contrast, Pore‐A is laterally connected to Pore‐B and Pore‐C through rectangular or pseudo‐square window apertures (Window‐AB and Window‐AC) along the *ab*‐plane. The LAD of these windows varies depending on the pillaring linker (Figures 1, S2, and S3)

Guest diffusion in these pillar‐layered MOFs is generally anisotropic, occurring predominantly along the *ab*‐plane due to the relatively larger windows (Window‐AB and Window‐AC) compared to the *c*‐axis Window‐AA.^[^ ^53^ ^]^ For instance, in the BP‐based Ni‐HBP structure, Window‐AB and Window‐AC exhibit LADs of 10.5 and 10.2 Å, respectively—substantially larger than the 6.7 Å of Window‐AA—rendering size‐selective diffusion along the *c*‐axis ineffective. Consequently, achieving selective adsorption requires blocking the *ab*‐plane channels and enforcing *c*‐axis transport through the narrower windows.

To reduce lateral channel dimensions, we introduced PZ, a shorter planar pillar, into the Ni‐HPZ structure. However, the crystal structure revealed that although PZ decreases the interlayer spacing, it does not completely obstruct the *ab*‐plane channels. The LADs of Window‐AB and Window‐AC reach up to 7.9 and 7.5 Å, respectively, due to additional variability in effective window size introduced by the rotational flexibility of the planar PZ pillar, which undermines size‐exclusion effects.^[^ ^36^, ^49^ ^]^

To overcome these limitations, we designed the Ni‐HDB framework using DABCO, a short, rigid, cylindrical 3D pillar. Ni‐HDB was synthesized via a de novo solvothermal reaction of Ni(NO_3_)_2_·6H_2_O, H_3_BTC, and DABCO in DMF at 70 °C for 3 days, yielding −100 µm‐sized single crystals suitable for X‐ray diffraction. The structure obtained was consistent with the previously reported post‐synthetic exchange product (Table S2).^[^ ^52^ ^]^

Ni‐HDB features a large Pore‐A (6.4 × 6.9 Å^2^), connected to another Pore‐A via a narrow elliptical Window‐AA (3.2 × 6.7 Å^2^) along the *c*‐axis (Figure 1). In contrast, Pore‐B and Pore‐C are substantially smaller (4.1 × 6.2 Å^2^ and 4.1 × 6.0 Å^2^, respectively) and are poorly accessible due to small window apertures (LAD of Window‐AB: 5.9 Å; Window‐AC: 5.7 Å). This effectively restricts guest diffusion to a one‐periodic channel along the *c*‐axis, mimicking a molecular bottleneck centered on Pore‐A and Window‐AA. The transformation from a three‐periodic to a one‐periodic diffusion network results in pronounced kinetic sieving. Moreover, the cylindrical DABCO pillar not only enforces channel confinement but also introduces a nonpolar, low‐dielectric pore environment that strengthens weak C─H⋯π and Van der Waals interactions with xylene molecules, varying subtly with isomer methyl group orientation.^[^ ^50^ ^]^ The structural stability of the framework, along with the non‐aromaticity of DABCO, further enhances the thermal and chemical robustness of Ni‐HDB under ambient conditions, a desirable attribute for industrial application.^[^ ^51^ ^]^

To demonstrate scalability—given the importance of bulk synthesis as a critical factor for practical applications^[^ ^54^, ^55^ ^]^—Ni‐HDB was synthesized under scaled‐up conditions (70 °C, 5 days), yielding 32.1 g of crystalline material with an 85.0% yield (see Figures S5–S13). Characterization via powder X‐ray diffraction (PXRD) and N_2_ sorption confirmed its phase purity and permanent porosity, which remained stable for over two months under ambient air (Figures S12 and S14). All subsequent adsorption and separation experiments were conducted using the bulk‐synthesized material.

**Figure 2:**
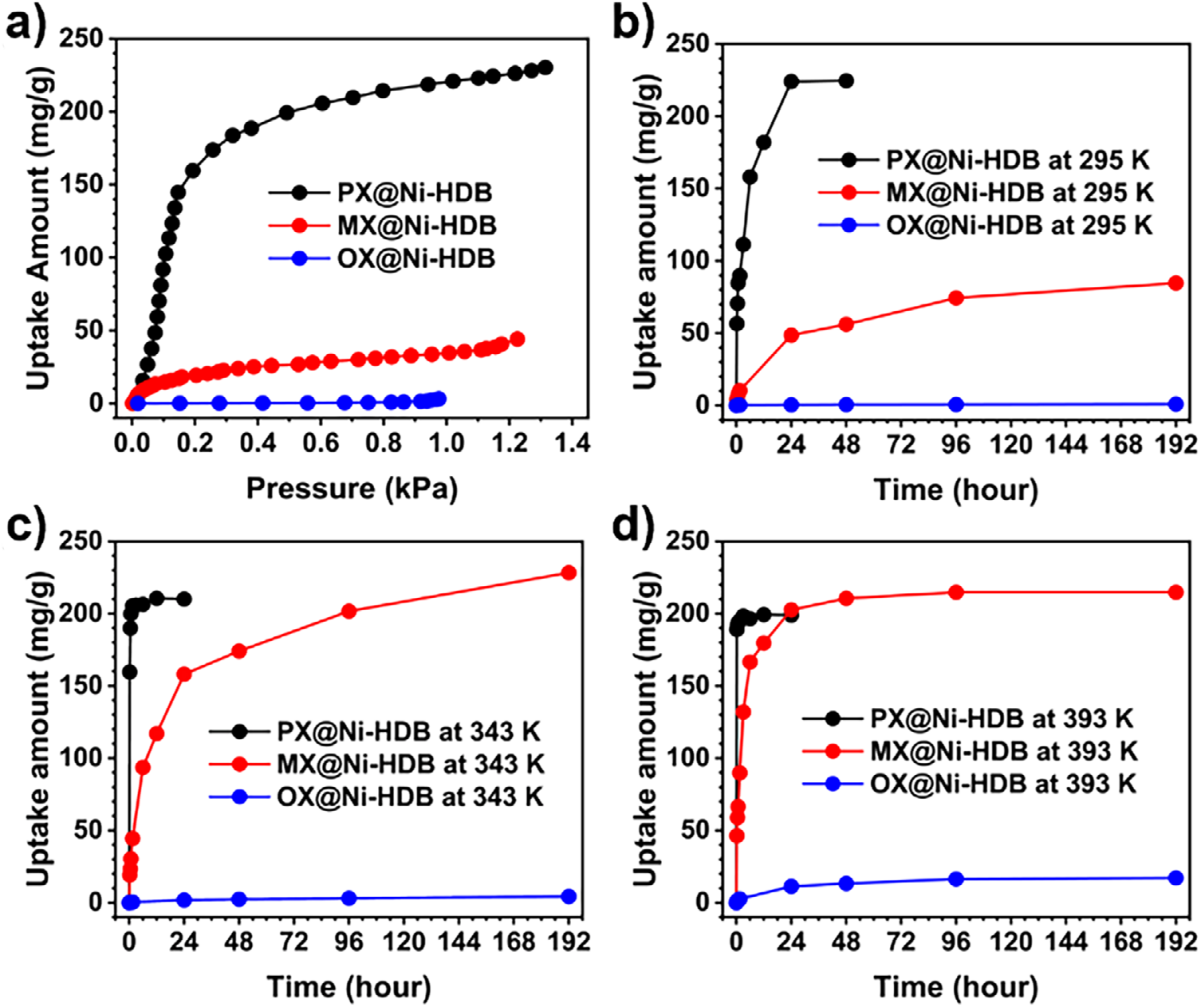
a) Single‐component xylene vapor adsorption isotherms on Ni‐HDB at 300 K. b)–d) Adsorption kinetics of Ni‐HDB from liquid‐phase single‐component xylene batch adsorption experiments at three different temperatures.

To evaluate the selective adsorption behavior of Ni‐HDB toward xylene isomers, single‐component vapor‐phase sorption experiments were conducted at 300 K (Figure 2a). Ni‐HDB displayed a high adsorption capacity for PX (230 mg g^−1^, equivalent to 0.82 PX molecules per BTC unit), a moderate uptake for MX (54.8 mg g^−1^), and negligible adsorption for OX (3.2 mg g^−1^). The steep uptake of PX highlights the compatibility between its molecular size and the static elliptical windows (Window‐AA), which act as molecular sieves. Although the structurally stable framework exhibits static aperture dimensions, local flexibility within the host allows for subtle dynamic adjustments during guest uptake. This flexibility arises from framework vibrations or localized deformation.^[^ ^26^, ^56^, ^57^, ^58^ ^]^ These effects permit accommodation of PX and, to some extent, MX, while still excluding the bulkier OX under ambient conditions.

To quantify the separation potential, ideal adsorbed solution theory (IAST) calculations were performed at 300 K using the IAST++ software (Figure S15).^[^ ^59^ ^]^ Based on the single‐component isotherms, calculated selectivities in equimolar binary mixtures were as follows: PX/OX = 846, MX/OX = 159, and PX/MX = 27.5 at 0.75 kPa. These values indicate strong kinetic discrimination, particularly between PX and OX, facilitated by the size‐selective diffusion barrier imposed by the narrow Window‐AA.

Liquid‐phase batch adsorption experiments were conducted to examine the separation behavior of Ni‐HDB under conditions relevant to industrial applications.^[^ ^10^, ^12^, ^49^ ^]^ The uptake kinetics were evaluated by soaking Ni‐HDB in each xylene isomer at various temperatures and analyzing the adsorbed amounts via ^1^H NMR after digestion (Figures 2b–d and S16–S18). At 295 K, PX was rapidly adsorbed, reaching saturation (224 mg g^−1^) within one day. MX showed slower uptake (84.7 mg g^−1^ after 8 days), while OX adsorption remained negligible (<1 mg g^−1^), confirming that the framework's sieving effect operates effectively under ambient conditions. The adsorption sequence—PX > MX >> OX—matches well with the vapor‐phase trend.

Temperature‐dependent experiments revealed that PX kinetics accelerated with increasing temperature: saturation was reached within 90 min at 343 K (205 mg g^−1^) and within 15 min at 393 K (189 mg g^−1^). In contrast, MX uptake increased significantly with temperature and time, eventually surpassing PX (228 mg g^−1^ at 343 K and 215 mg g^−1^ at 393 K after extended soaking). The adsorption kinetics are accelerated at elevated temperatures due to the increased likelihood of framework expansion from thermal lattice vibrations, which facilitate faster diffusion.^[^ ^60^, ^61^, ^62^ ^]^ OX, however, remained largely excluded at both elevated temperatures (4.5 mg g^−1^ at 343 K, 17.5 mg g^−1^ at 393 K). These findings suggest a temperature‐driven shift in the governing mechanism—from kinetic control at lower temperatures, favoring PX due to size‐exclusion, to thermodynamic control at higher temperatures, favoring MX due to stronger host–guest interactions. Notably, the maximum adsorption capacities of Ni‐HDB for PX (224 mg g^−1^) and MX (228 mg g^−1^) exceed those of state‐of‐the‐art adsorbents, including Mn‐dhbq (208/205 mg g^−1^),^[^ ^23^ ^]^ HIAM‐203 (199/159 mg g^−1^),^[^ ^24^ ^]^ Cu‐metallocycle (140/117 mg g^−1^),^[^ ^8^ ^]^ and MAF‐89 (212/186 mg g^−1^) (Figure S19).^[^ ^26^ ^]^

**Figure 3:**
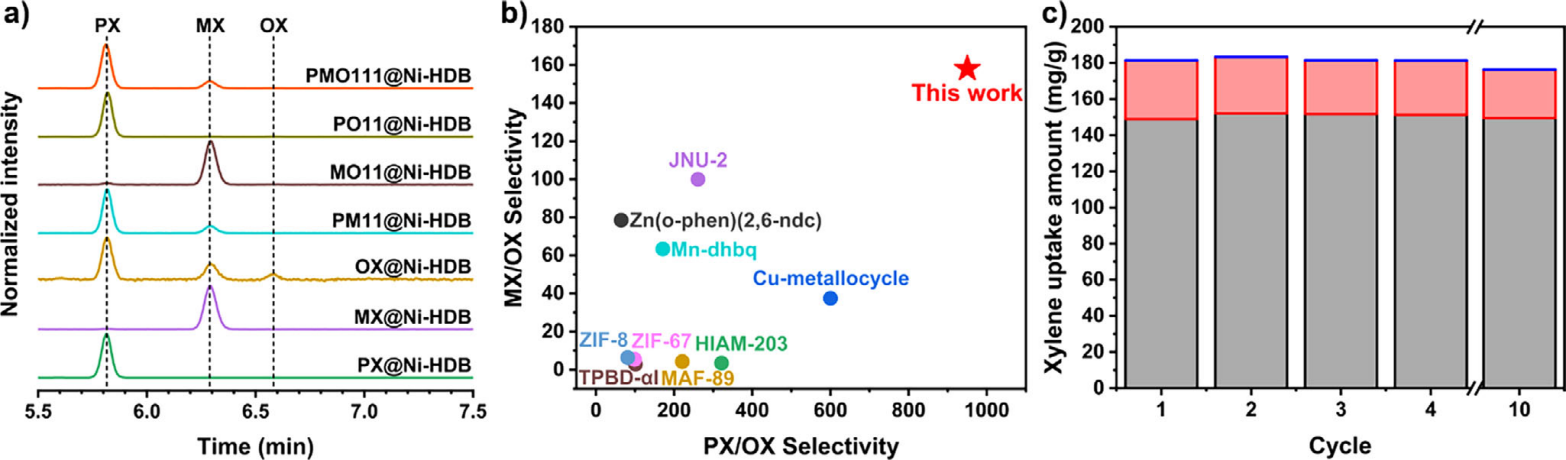
a) Result of gas chromatographic experiments quantifying the xylene content adsorbed in Ni‐HDB from single‐ and multi‐component equimolar xylene isomer solutions under ambient conditions (295 K) for 1 day; all peak intensities were normalized. b) Comparison of PX/OX and MX/OX selectivities for Ni‐HDB and previously reported potential. For each reported material, only the highest selectivity values were included. c) Xylene uptake during the recyclability test of Ni‐HDB. The relative uptake of each xylene isomers was monitored (gray: PX, red: MX, blue: OX).

The liquid‐phase single‐component adsorption experimental results suggest that Ni‐HDB not only has a high capacity for PX but also can effectively discriminate between xylene isomers with remarkable efficiency and selectivity under ambient conditions (Figures 3a and S20). Even when soaked in pure OX (≥99.0% purity), trace amounts of PX and MX impurities were preferentially adsorbed over OX, illustrating the framework's strong exclusion of OX. To simulate practical separation scenarios, Ni‐HDB was tested in equimolar binary liquid mixtures of xylene isomers. Gas chromatography analysis after 1‐day adsorption at 295 K revealed exceptional selectivities: 951 (PX/OX), 158 (MX/OX), and 4.99 (PX/MX) for binary mixtures (Figures 3b, S21, and S22). To the best of our knowledge, Ni‐HDB exhibits the highest reported selectivities for PX/OX and MX/OX, surpassing all previously reported adsorbents (Figure 3b; Tables S3 and S4).^[^ ^7^, ^8^, ^9^, ^23^, ^24^, ^25^, ^26^, ^27^ ^]^ The outstanding separating performance of Ni‐HDB in binary equimolar mixture experiment encouraged us to further evaluate its efficiency in a ternary equimolar mixture (1:1:1) (Figure S23). A similar experiment, conducted under the same conditions as the binary test, resulted in a selectivity ratio of 717/141/1 for PX/MX/OX, which is consistent with the trend observed in the binary mixture, despite slight variations in selectivity. The separation of PX from complex industrial mixtures, including ethylbenzene (EB), is of significant practical relevance. Accordingly, we conducted additional adsorption experiments using a representative industrial xylene mixture (PX:MX:OX:EB = 22:50:22:6)^[^ ^63^ ^]^ at 295 K. Gas chromatographic analysis revealed that Ni‐HDB exhibited the highest selectivity toward EB, with an EB/PX selectivity of 2.79 and an exceptionally high EB/OX selectivity of 1943—substantially exceeding those of PX/OX (695) and MX/OX (130) (see Figure S24). This preferential uptake of EB can be attributed to both kinetic and thermodynamic factors. While the MIN‐2 value of EB (6.53 Å) is comparable to that of PX (6.51 Å), facilitating diffusion, its higher binding energy (63.46 kJ mol^−1^) relative to PX (59.40 kJ mol^−1^)—as calculated using density functional theory (DFT) in VASP^[^ ^64^, ^65^, ^66^ ^]^ based on optimized structures of PX‐ and EB‐loaded Ni‐HDB—enhances its thermodynamic affinity (see Figures S25 and S26; Table S5). Although Ni‐HDB cannot selectively separate PX from EB, it can still play a valuable role in multi‐step separation strategies,^[^ ^67^ ^]^ particularly given EB's relatively low concentration in industrial feedstocks and its catalytic convertibility into other xylene isomers.^[^ ^68^ ^]^

To mimic practical adsorption–desorption conditions, PX‐loaded Ni‐HDB was treated with para‐diethylbenzene (p‐DEB), a commonly used industrial desorbent. When 10 mg of PX‐loaded Ni‐HDB was immersed in 1 mL of p‐DEB at 120 °C for 1 day, the PX loading decreased from −0.80 to 0.067 molecules per BTC, yielding an extraction efficiency of 92%. Concurrently, 0.36 molecules of p‐DEB per BTC were adsorbed (Figure S27). Furthermore, since reusability is a key factor for practical application—especially in reducing the amount of absorbent used—a recyclability test was conducted with Ni‐HDB. It maintained both its selectivity and capacity for the xylene ternary equimolar mixture (1:1:1) over 10 cycles (Figures 3c and S28). The PXRD patterns and porosity of Ni‐HDB before and after the recyclability test were nearly identical, indicating excellent framework stability and structural integrity under working conditions (Figure S29). This stability underpins the consistent cyclic performance of Ni‐HDB, highlighting its potential for repeated use in xylene separation processes.

Unlike at ambient temperature, the xylene adsorption behavior of Ni‐HDB at elevated temperatures revealed a reversal in the adsorption capacities of PX and MX, observed after sufficient saturation time at 393 K (Figure 2). This behavior is closely associated with thermodynamic effects; the distinctive interactions between xylene isomers and the framework within confined spaces significantly influence the preferred adsorbed molecule. To elucidate the reversed adsorption trend of PX and MX at higher temperatures, the binding energies (per molecule) of optimized structures of PX‐, MX‐, and OX‐loaded Ni‐HDB were calculated using DFT at 0 K, yielding the following values: PX@Ni‐HDB = 59.40 kJ mol^−1^, MX@Ni‐HDB = 61.22 kJ mol^−1^, and OX@Ni‐HDB = 53.56 kJ mol^−1^ (Figures S25, S30, and S31; Table S5). These results indicate that MX is thermodynamically favored over PX, and both are significantly more favorable than OX. The simulations also revealed consistent unit cell expansion upon xylene adsorption, particularly in PX and MX‐loaded structures, in alignment with experimentally observed PXRD shifts (Figure S32). Especially, the (−1 2 0) peak at 2θ = 10.608° and the (0 0 1) peak at 2θ = 12.633° both shifted to lower angles, indicating a unit cell enlargement—an effect not observed for OX, which is experimentally excluded from Ni‐HDB. These computational calculation results support the selective xylene adsorption behavior of Ni‐HDB: at ambient temperature (295 K), PX is kinetically favored due to a sieving effect within the confined one‐periodic channel, whereas at elevated temperature (393 K), MX becomes thermodynamically preferred as the diffusion barrier through the narrow Window‐AA is overcome. In contrast, OX, being the larges isomer, remains disfavored in both regimes. The combination of high kinetic barriers (imposed by the narrow Window‐AA) and differential binding affinities accounts for the experimentally observed temperature‐dependent selectivity trends: PX is favored under ambient conditions due to kinetic factors, while MX becomes preferred at higher temperatures due to thermodynamic stability. OX, being the largest isomer, is disfavored in both respects.

To validate our diffusion control strategy, we synthesized two isoreticular analogues—Ni‐HBP and Ni‐HPZ—bearing long‐and‐planar (BP) and short‐and‐planar (PZ) pillaring ligands, respectively, and systematically compared their xylene separation performances (Figures S33 and S34). All three MOFs (Ni‐HBP, Ni‐HPZ, and Ni‐HDB) possess the same primary *c*‐axis elliptical windows (Window‐AA, −6.7 Å). However, the dimensions of interlayer pores and lateral windows differ significantly due to the geometry and length of the pillaring ligands (Figures 1, S2, and S3).

Ni‐HBP, incorporating the long BP pillar, exhibits large lateral window apertures (LADs of Window‐AB and Window‐AC: 10.5 and 10.2 Å), which allow nonselective diffusion of xylene isomers along the *ab*‐plane. Consequently, Ni‐HBP exhibits negligible size‐sieving behavior. In ternary liquid‐phase batch adsorption experiments, it preferentially adsorbs OX over PX and MX, resulting in selectivities of OX/PX/MX = 2.26/1.34/1 (Figure S35). This trend suggests that, in the absence of diffusion constraints, thermodynamic factors dominate and selectivity is lost. In contrast, Ni‐HPZ incorporates a shorter, planar PZ pillar, which partially reduces interlayer spacing. This geometric modification narrows lateral windows (LADs of Window‐AB and Window‐AC: 7.9 and 7.5 Å), enhancing the kinetic sieving effect to some extent. However, due to the rotational freedom of the 2D planar PZ pillar, the effective size and shape of the windows are dynamic and poorly defined, allowing partial diffusion of larger isomers. Consequently, Ni‐HPZ exhibits moderate PX selectivity but still performs substantially worse than Ni‐HDB. In ternary mixtures, its selectivities were PX/MX/OX = 3.55/1.27/1 (Figure S36). These results confirm that complete blockage of *ab*‐plane channels and restriction to a narrow, one‐periodic *c*‐axis pathway—achievable only through short cylindrical 3D pillars like DABCO—is essential for achieving ultrahigh selectivity for PX and EB, while explicitly excluding OX.

## Conclusion

This work demonstrates a rational channel engineering approach to overcome the long‐standing challenge of xylene isomer separation. By introducing a rigid cylindrical DABCO pillar into the isoreticular Ni‐based MOF framework [Ni_3_(H_1.5_BTC)_2_(BTC)(Pillar)_3_], we precisely restricted guest diffusion to one‐periodic channels aligned along the *c*‐axis. This configuration allowed full exploitation of static elliptical windows for size‐sieving, leading to record‐high liquid‐phase selectivities of 1943 (EB/OX), 951 (PX/OX) and 158 (MX/OX), along with high adsorption capacities, while explicitly excluding OX under ambient conditions. Systematic comparisons with isoreticular analogues (Ni‐HBP and Ni‐HPZ) confirmed the critical role of 3D pillar geometry in blocking lateral diffusion and enhancing discrimination efficiency. Complementary DFT calculations supported the observed separation behavior, revealing a kinetic preference for PX and EB under ambient conditions, a thermodynamic preference for MX at elevated temperatures, and the exclusion of OX in both cases due to steric mismatch. The Ni‐HDB framework also demonstrated excellent chemical and structural stability under ambient conditions and retained performance over 10 adsorption–desorption cycles. These features underscore its potential as a practical, energy‐efficient adsorbent for industrial xylene separation.

More broadly, this study illustrates how fine‐tuned control of channel dimensionality and geometry through isoreticular design can be leveraged to address key challenges in hydrocarbon separations. The principles developed here may be readily extended to other molecular sieving applications where subtle differences in molecular size and shape must be resolved with high precision.

## Conflict of Interests

The authors declare no conflict of interest.

## Supporting information



Supporting Information

Supporting Information

## Data Availability

The data that support the findings of this study are available from the corresponding author upon reasonable request.
