# Supplementary material for: Highly Selective Adsorption of Para‐Xylene, Ethylbenzene, and Explicit Exclusion of Ortho‐Xylene from Xylene Isomers Using a Pillar‐Layered MOF with Tuned Pore Channels
Source: Angew Chem Int Ed Engl. 2025 Jul 18;64(39):e202512244. doi: 10.1002/anie.202512244 (PMC12455432; doi:10.1002/anie.202512244)
Supplement: Supplementary file 1 — Supporting Information [file ANIE-64-e202512244-s002.docx]

Highly Selective Adsorption of para-Xylene and Ethylbenzene and Explicit Exclusion of ortho-Xylene from Xylene Isomers Using a Pillar-Layered MOF with Tuned Pore Channels

Supporting Information
©Wiley-VCH 2019
69451 Weinheim, Germany

**Abstract:** Xylene isomer separation is a long-standing challenge due to the nearly identical properties of para-xylene (PX), meta-xylene (MX), ortho-xylene (OX), and ethylbenzene (EB). Here, we report a rationally designed pillar-layered metal–organic framework (MOF), Ni-HDB, incorporating a cylindrical 1,4-diazabicyclo[2.2.2]octane (DABCO) pillar that blocks lateral channels and directs molecular transport through elliptical windows (3.2 × 6.7 Å²). These apertures closely match the dimensions of PX and EB, enabling kinetic sieving. As a result, Ni-HDB exhibits high selectivity for PX and EB, moderate selectivity for MX, and exclusion of OX under ambient conditions. It achieves record liquid-phase selectivities for EB/OX (1943), PX/OX (951), and MX/OX (158), along with high PX and MX adsorption capacities. Comparative studies with isoreticular analogues confirm that DABCO-driven confinement is key to enhancing size-based selectivity. Density functional theory calculations indicate kinetic preference for PX and EB, thermodynamic favorability for MX, and exclusion of OX. Ni-HDB also shows excellent thermal and structural stability, with no performance loss over ten cycles. These results highlight the importance of channel geometry in MOFs and provide a framework for developing next-generation adsorbents for energy-efficient hydrocarbon separations.

DOI: 10.1002/anie.2025XXXXX

Table of Contents

Experimental Procedures 2

Materials and General Procedures 2

Preparation of MOFs 2

Synthesis of Ni-HDB 2

Bulk-Scale Synthesis of Ni-HDB 2

Synthesis of Ni-HBP 2

Synthesis of Ni-HPZ 2

Crystallographic Data Collection and Structure Refinement 2

Crystal Structure of Ni-HDB 2

Pores and Windows in [Ni_3_(H_1.5_BTC)_2_(BTC)(Pillar)_3_] Structure 2

Gas Sorption 3

Vapor Sorption of Xylene Isomers and IAST Calculation 3

Liquid Sorption of Xylene Isomers 3

Selective Adsorption Behavior of Ni-HDB under an Industrial Composition Mixture 3

Liquid-phase Adsorption Kinetics 3

Calculation of Selectivity 3

Recyclability Test 4

Computational Details 4

Supporting Figures S1–S36 5

Supporting Tables S1–S5 41

References 46

Author Contributions 47

Experimental Procedures

**Materials and General Procedures**

All reagents and solvents were purchased from commercial suppliers and used without further purification. The xylene isomers—*para*-xylene (PX), *meta*-xylene (MX), and *ortho*-xylene (OX)—as well as ethylbenzene (EB), were obtained from Sigma-Aldrich (purity > 99%). Product and batch numbers were as follows: PX (95682/STBJ9036), MX (95672/STBK1649), OX (95662/STBK9993), and EB (E12508/SHBN1509). Elemental analysis (EA; C, H, N) was performed using a Flash 2000 analyzer at the Central Research Facilities of UNIST, Republic of Korea. Powder X-ray diffraction (PXRD) data were collected at room temperature on a Bruker D2 Phaser and Rigaku SmartLab diffractometer with a 2θ step size of 0.02°. Simulated PXRD patterns were generated using Materials Studio software^[S1]^ based on single-crystal structures. Thermogravimetric analysis (TGA) was conducted using a TA Instruments Q-600 system at a heating rate of 5 °C/min under nitrogen flow. Proton nuclear magnetic resonance (^1^H NMR) spectra were recorded using a 400 MHz FT-NMR spectrometer (Agilent).

**Preparation of MOFs**

**Synthesis of Ni-HDB**

In a 20 mL vial, 22.4 mg (0.200 mmol) of 1,4-diazabicyclo[2.2.2]octane (DABCO) was dissolved in 1.0 mL of *N,N*-dimethylformamide (DMF). Separately, 42.0 mg (0.200 mmol) of benzene-1,3,5-tricarboxylic acid (H_3_BTC) dissolved in 1.0 mL of DMF was added dropwise to the wall of the vial. Then, 58.0 mg (0.200 mmol) of Ni(NO_3_)_2_·6H_2_O in 1.0 mL of DMF was added. Finally, 3.0 mL of methanol (MeOH) was introduced and the mixture was shaken until all solids dissolved. The sealed vial was heated at 70 °C for 3 days. Upon cooling to room temperature, green block-shaped crystals were collected, washed, and activated by soaking in fresh DMF (2×15 mL) and dichloromethane (DCM) (2×15 mL) over two days, followed by drying under dynamic vacuum at 120 °C for 24 hours. EA of Ni-HDB: [Ni_3_(H_1.5_BTC)_2_(BTC)(DABCO)_2.79_(DMF)_0.21_(H_2_O)_0.21_]∙18H_2_O (C_44.37_H_83.37_N_5.79_O_36.42_Ni_3_, formula weight (fw) = 1456.82 g/mol). Found (calcd): C, 36.20% (36.58%); H, 5.81% (5.77%); N, 5.62% (5.57%). Yield of the activated Ni-HDB: 68.0 mg (90.1%).

**Bulk-Scale Synthesis of Ni-HDB**

In a 3 L Erlenmeyer flask, 13.5 g (120 mmol) of DABCO was dissolved in 250 mL of DMF. Separately, 21.0 g (100 mmol) of H_3_BTC and 29.5 g (101 mmol) of Ni(NO_3_)_2_·6H_2_O were each dissolved in 250 mL of DMF and sequentially added. After shaking for 10 minutes, 1 L of MeOH was added, and the mixture was further shaken until all solids dissolved. The solution was heated at 70 °C for 5 days and shaken once daily after 48 hours. Upon cooling, crystals were harvested and washed with DMF and DCM (each twice, 200 mL), and activated as described above. EA of Ni-HDB in Bulk Scale: [Ni_3_(H_1.5_BTC)_2_(BTC)(DABCO)_2.88_(DMF)_0.12_(H_2_O)_0.12_]∙14H_2_O (C_44.64_H_75.64_N_5.88_O_32.24_Ni_3_, fw = 1386.66 g/mol). Found (Calcd): C, 38.68% (38.67%); H, 5.98% (5.50%); N, 5.98% (5.94%). Yield of the activated Ni-HDB: 32.1 g (85.0%).

**Synthesis of Ni-HBP**

Approximate 50 μm-sized crystals of Ni-HBP with the formula unit [Ni_3_(H_1.5_BTC)_2_(BTC)(BP)_3_] (where BP is 4,4'-bipyridine) were prepared according to a previously reported procedure with slight modifications.^[S2]^ Specifically, 37.8 mg (0.130 mmol) of Ni(NO_3_)_2_·6H_2_O, 23.2 mg (0.110 mmol) of H_3_BTC, and 17.2 mg (0.110 mmol) of BP were dissolved in 4 mL of DMF and 4 mL of MeOH. The mixture was heated at 70 °C for 2 days, producing cyan plate-shaped crystals. The product was activated using the same procedure as Ni-HDB. Yield: 24.1 mg (96.4%).

**Synthesis of Ni-HPZ**

Ni-HPZ, with the formula unit [Ni_3_(H_1.5_BTC)_2_(BTC)(PZ)_3_] (where PZ is pyrazine), was prepared according to a previously reported procedure with slight modifications.^[S3]^ Approximately 100 mg of Ni-HBP crystals (pre-soaked in DMF) were transferred into 20 mL of a 2.0 M PZ solution in DMF and heated at 100 °C for 1 month. The resulting cyan crystals were washed, activated as described above, and used without further purification. Successful exchange of BP with PZ was confirmed by ^1^H NMR.

**Crystallographic Data Collection and Structure Refinement**

The diffraction data of Ni-HDB crystal coated with Paratone oil were measured at 100 K using synchrotron radiation (*λ* = 0.70000 Å) and a Rayonix MX225HS detector at 2D SMC with a Si(111) double-crystal monochromator at the Pohang Accelerator Laboratory, Republic of Korea. Data collection was performed using PAL BL2D-SMDC software,^[S4]^ and cell refinement, data reduction, and absorption correction were carried out using HKL3000sm software (ver. 703r).^[S5]^ The crystal structure was solved by direct methods and refined by full-matrix least-squares calculations using the SHELX package.^[S6]^

**Crystal Structure of Ni-HDB**

[Ni_3_(H_1.5_BTC)_2_(BTC)(DABCO)_3_] (C_45_H_48_N_6_O_18_Ni_3_), fw = 1137.02 g·mol^–1^, crystallizes in the hexagonal *P*-62*m* space group. Each Ni^2+^ ion is coordinated to the carboxylate group of one bidentate BTC^3−^ and two monodentate H_1.5_BTC^1.5−^ ligands. The three carboxylate hydrogen atoms in each H_1.5_BTC^1.5−^ ligand, with an occupancy of 0.5, are assumed to be shared with three adjacent H_1.5_BTC^1.5−^ ligands, resulting in the formation of a two-periodic layer. The neutral, cylindrical DABCO pillars bridges the Ni centers of adjacent two-periodic Ni-BTC layers in an eclipsed fashion, giving rise to a three-periodic structure with a 3,5-c **hms** net topology. All non-hydrogen atoms were refined anisotropically. Hydrogen atoms, except those on the carboxylate groups of the H_1.5_BTC^1.5−^ ligands, were placed in calculated positions with isotropic displacement parameters (U(H) = 1.2U(C)) and refined using a riding model. Final refinement was carried out using the SQUEEZE option in PLATON^[S7]^ to account for electron density from disordered solvent molecules (721 Å^3^, 43.7% of the unit cell volume; 211 electrons per unit cell). The refinement converged with final values of *R*1 = 0.0917 and *wR*2 = 0.2275 for 1854 reflections with *I* > 2*σ*(*I*); *R*1 = 0.0920 and *wR*2 = 0.2300 for all 1868 reflections. The largest residual electron density peak and hole were +2.993 and –2.406 e·Å^−3^, respectively.

A summary of the crystallographic data is provided in Table S2. Crystallographic data for Ni-HDB have been deposited with the Cambridge Crystallographic Data Centre (CCDC 2451628) and can be obtained free of charge from [www.ccdc.cam.ac.uk/conts/retrieving.html](http://www.ccdc.cam.ac.uk/conts/retrieving.html) or by contacting the CCDC at 12 Union Road, Cambridge CB2 1EZ, UK.

**Pores and Windows in [Ni_3_(H_1.5_BTC)_2_(BTC)(Pillar)_3_] Structure**

The [Ni_3_(H_1.5_BTC)_2_(BTC)(Pillar)_3_] structure contains three distinct types of pores. Pore-A is vertically interconnected with other Pore-A units through elliptical Window-AA, forming a one-periodic channel along the *c*-axis. It is also connected to two Pore-B units and one Pore-C unit along the *ab*-plane, thereby establishing a three-periodic channel system in five directions (as shown in Figure S4). Window-AA consistently exhibits the largest aperture diameter (LAD), approximately 6.6–6.7 Å, regardless of the pillar ligand employed.

In contrast, the vertical extension of channels through Pore-B and Pore-C is blocked by BTC ligands, restricting them to form only three-directional, two-periodic channels connected to adjacent Pore-A units in the horizontal direction between Ni–BTC layers. The coordination modes of BTC ligands differ between Pore-B and Pore-C: in Pore-B, all BTC ligands coordinate to Ni^2+^ ions in a monodentate fashion, whereas in Pore-C, they coordinate in a bidentate manner. As a result, the ratio of Pore-A, Pore-B, and Pore-C is 3:2:1.

**Gas Sorption**

Gas sorption isotherms were measured using a BELSORP-Max instrument (BEL Japan, Inc.) via a standard volumetric technique up to the saturation pressure. N_2_ (99.9999%) adsorption and desorption isotherms were collected at 77 K. Brunauer–Emmett–Teller (BET) surface area analysis was performed using SESAMI software,^[S8]^ based on both GCMC-simulated and experimental N_2_ adsorption isotherms. The adsorption data were converted into BET plots, and linear regions containing at least four data points that met all four consistency criteria were selected for surface area calculation.

**Vapor Sorption of Xylene Isomers and IAST Calculation**

Xylene vapor sorption measurements were performed using a BELSORP-Max at 300 K, with the bath temperature maintained by a recirculating heater/cooler. For repeated measurements, samples were regenerated by degassing under vacuum at 120 °C for several hours.

Ideal Adsorbed Solution Theory (IAST) calculations were performed over a pressure range of 0.05–1.4 kPa at 300 K for an equimolar composition, using best-fit models within the IAST++ software package.^[S9]^ The Henry isotherm model, Langmuir–Freundlich isotherm model, and dual-site Langmuir isotherm model were employed to fit the adsorption isotherms of OX, PX, and MX, respectively, on Ni-HDB. These models are described as follows:

Linear (Henry): m(P) = kP

Langmuir–Freundlich (LF): m(P) = q·[(kP)^n^ / {1 + (kP)^n^}]

Dual-Site Langmuir (DSL): m(P) = q_1_[k_1_P / (1 + k_1_P)] + q_2_[k_2_P / (1 + k_2_P)]

where m represents the total adsorption amount (mmol/g), P is the total pressure of the bulk vapor at adsorption equilibrium, k is the fitted constant, q denotes the adsorption capacity (mmol/g), n is the Freundlich exponent, and q_1_, q_2_, k_1_, and k_2_ correspond to the adsorption capacities and fitted constants for sites 1 and 2, respectively.

**Liquid Sorption of Xylene Isomers**

In liquid-phase batch sorption experiments with xylene isomers, approximately 10 mg of activated Ni-HDB crystals were immersed in 1 mL of each xylene isomer at 295 K, 343 K, and 393 K, respectively. The recovered crystals were dried on filter paper for 10 minutes to remove any residual xylene on the outer surface. To analyze the uptake amount of xylene isomers, two methods were used: ^1^H NMR spectroscopy (Method I) for general uptake analysis, and gas chromatography (GC) (Method II) for precise calculation of selectivity under different conditions.

Method I: The xylene-loaded MOF crystals were dissolved in a solvent mixture consisting of 0.06 mL of concentrated DCl and 0.72 mL of dimethyl sulfoxide-*d_6_* (DMSO-*d_6_*). The relative intensities of the proton peaks corresponding to the xylene isomers, compared to those of the BTC linker in the ^1^H NMR spectrum, were used to quantify the adsorbed xylene isomer content.

Method II: The xylene-loaded crystals were dissolved in 1 mL of H_2_O, followed by the addition of 1 mL of CHCl_3_. The mixture was shaken several times to extract the xylene isomers into the organic layer. The collected organic phase was analyzed by GC to determine the relative amounts of the xylene isomers. GC analyses were performed using an Agilent 7890A/5975C gas chromatograph equipped with a mass spectrometry detector and helium as the carrier gas. The column used was a WCOT CP-Xylenes column (Part No. CP7426) with dimensions of 0.32 mm × 50 m. The GC peak areas were used to calculate the selectivity.

**Selective Adsorption Behavior of Ni-HDB under an Industrial Composition Mixture**

In industrial xylene separation processes, EB is also a crucial component. Therefore, to evaluate the performance of Ni-HDB in the presence of EB, we conducted additional liquid-phase batch sorption experiments using an industrial composition mixture (PX:MX:OX:EB = 22:50:22:6)^[S14]^ at room temperature for 1 day.

**Liquid-phase Adsorption Kinetics**

The experimental procedure for measuring liquid-phase adsorption kinetics is essentially identical to the batch sorption protocol described (Method I). Briefly, approximately 10 mg of activated Ni-HDB crystals were immersed in 1 mL of each xylene isomer at 295 K, 343 K, or 393 K. At predetermined time intervals, the xylene-soaked crystals were recovered and gently dried under ambient conditions for 10 minutes to remove any residual surface xylene. The amount of xylene adsorbed was subsequently quantified by ^1^H NMR spectroscopy.

**Calculation of Selectivity**

To calculate the adsorption selectivity for mixtures of xylene isomers on Ni-HDB, the selectivity (*S*_ij_) is defined as:

*S*_ij_ = (*x*_i_/*x*_j_)/(*y*_i_/*y*_j_)

where *x*_i_ and *x*_j_ represent the adsorption amounts of components *i* and *j*, respectively, on the sample under equilibrium conditions (typically expressed in mmol/g). *y*_i_ and *y*_j_​ are the corresponding mole fractions in the mixture.

IAST-based adsorption selectivities were calculated for PX/OX (1:1 molar ratio), MX/OX (1:1), and PX/MX (1:1) mixtures, assuming mole fractions of *y*_1_ = 0.5 and *y*_2_ = 1 − *y*_1_ = 0.5, over a pressure range of 0.05–1.4 kPa at 300 K. Solution-phase selectivities for equimolar binary or ternary mixtures of xylene isomers were similarly calculated based on the relative uptake amounts of components *i* and *j* in the adsorbent, as determined from the peak areas in GC chromatograms.

**Recyclability Test**

A total of 200 mg of activated Ni-HDB crystals was soaked in 20 mL of a ternary equimolar (1:1:1) xylene isomer solution at 295 K for 1 day. After soaking, the xylene-loaded crystals were harvested, placed on filter paper, and air-dried for 30 minutes. A portion of the sample (approximately 10 mg) was taken for ^1^H NMR and GC analysis. The remaining crystals were reactivated by placing them in a vacuum oven at 295 K for 1 day, followed by heating at 120 °C for an additional day. The reactivated crystals were then re-soaked in 20 mL of the same xylene isomer solution. This cycle was repeated 10 times.

**Computational Details**

Density functional theory (DFT) calculations were performed using the Vienna Ab-initio Simulation Package (VASP),^[S10–S12]^ employing the PBE functional.^[S13]^ The plane-wave cutoff was set to 400 eV, and *k*-point sampling was conducted using a 2×2×1 Γ-centered Monkhorst–Pack mesh. Various initial structures of xylene-loaded Ni-HDB were generated based on experimental X-ray crystal structures, placing xylene molecules in plausible positions (Figures S26–S28). A supercell consisting of two Ni-HDB layers connected by DABCO, with vacuum spacing along the *c*-axis, was used to investigate xylene loading. Three xylene molecules were included per unit cell to preserve crystal symmetry during geometry and cell optimization.

Based on the optimized structures, the binding energy per molecule (B.E.) was calculated using the equation:

B.E. = (*E*_Xylene@Ni-HDB_ – *E*_Ni-HDB_ – 3*E*_Xylene_)/3,

where *E*_Xylene@Ni-HDB_ and *E*_Ni-HDB_ are the total energies of the xylene-loaded and unloaded Ni-HDB structures, respectively, with fully optimized cell parameters, and *E*_Xylene_ is the energy of a single xylene molecule within the unit cell of Xylene@Ni-HDB. Table S5 presents the optimized cell parameters, interlayer distances, and total energies for each xylene-loaded Ni-HDB system.


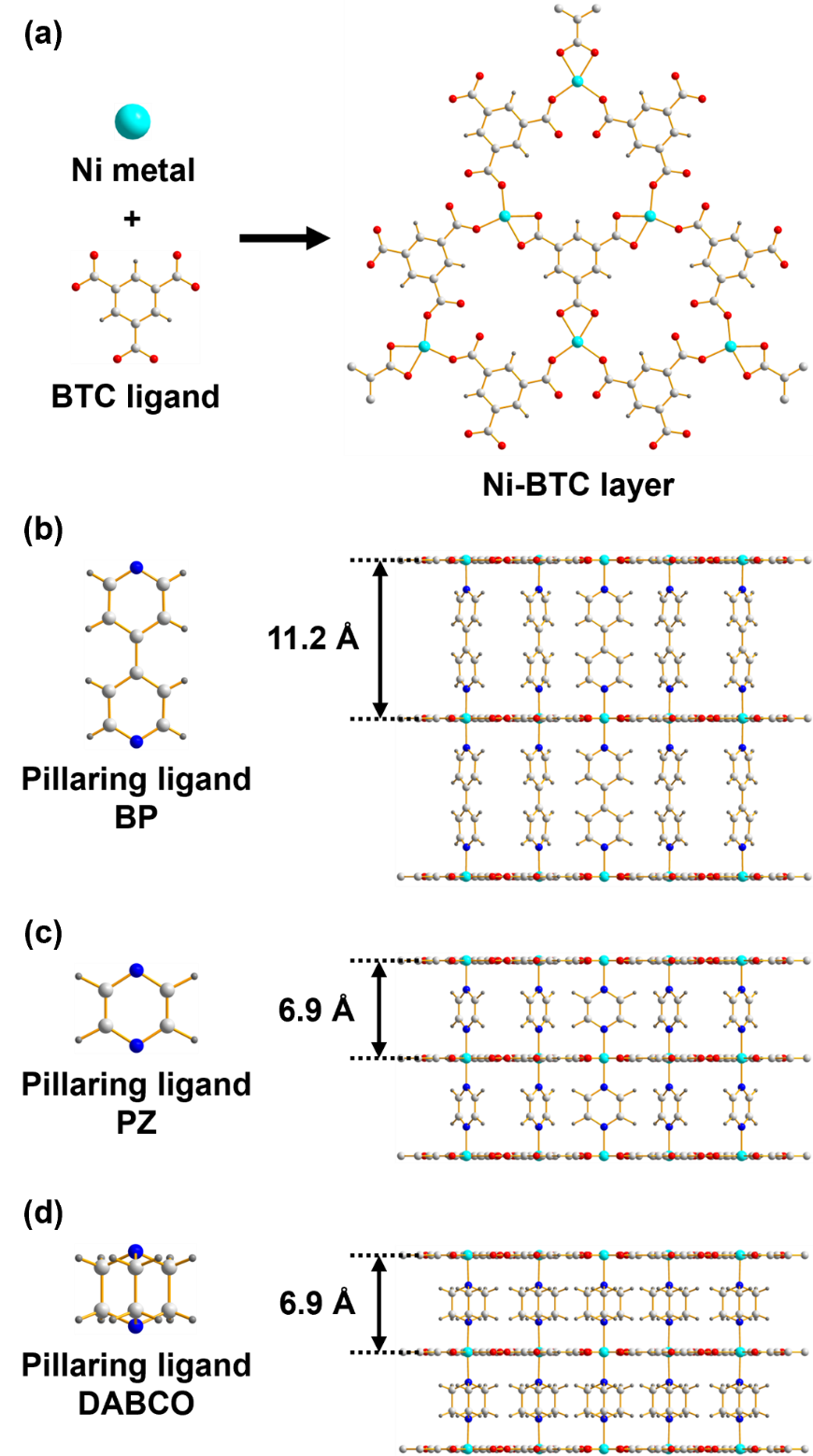


**Figure S1.** (a) Formation of two-periodic metal–organic layers. (b–d) Side views of isoreticular pillar-layered MOFs with different pillar lengths and geometries: Ni-HBP, Ni-HPZ, and Ni-HDB. Disorder is omitted for clarity.


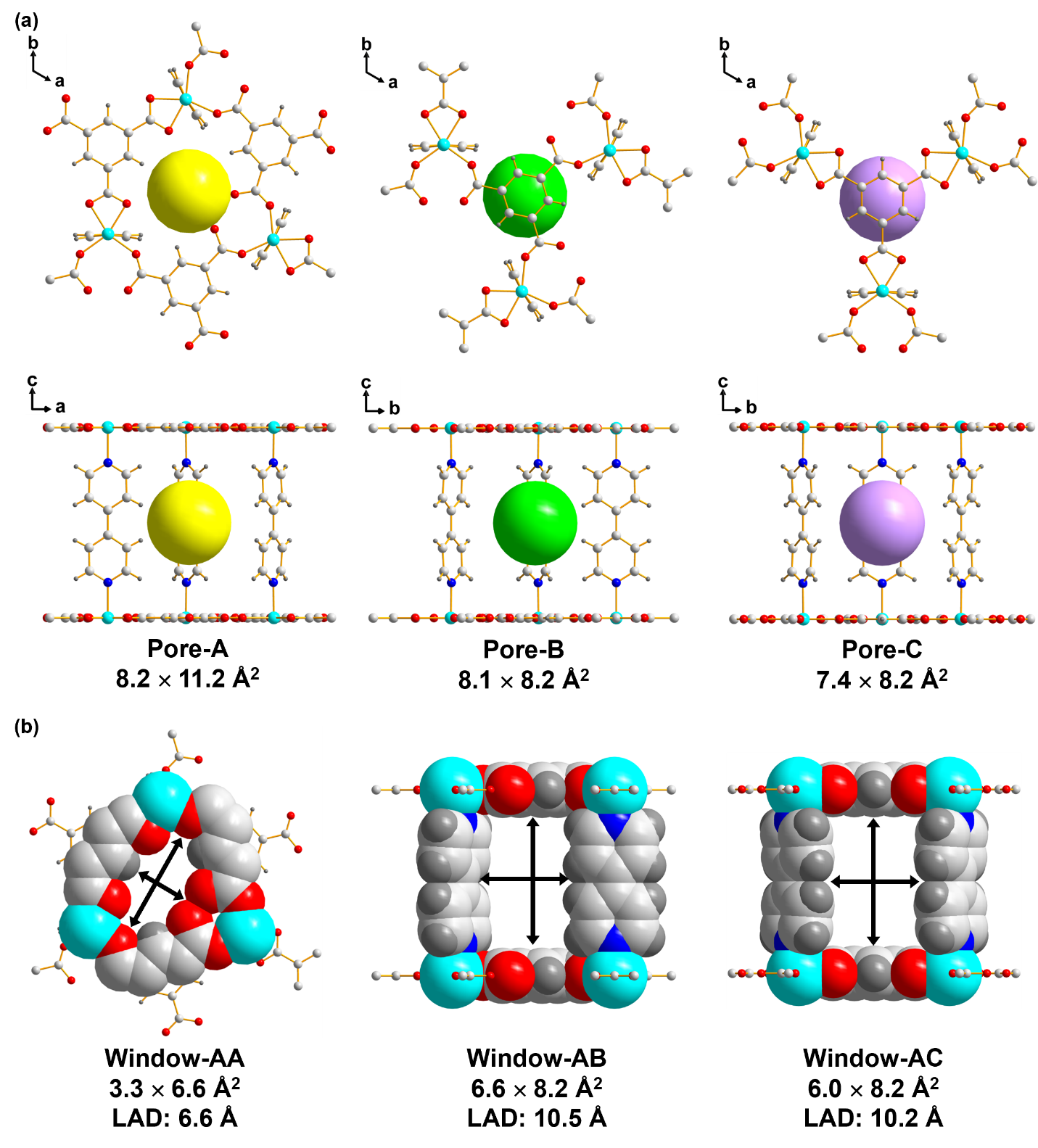


**Figure S2.** Pores and windows in Ni-HBP. (a) Graphical representations of Pore-A, Pore-B, and Pore-C, shown in top view (top) and side view (bottom). (b) Graphical representations of individual windows. The LAD was calculated as the maximum diagonal distance within each window, considering potential rotation of the pillar.


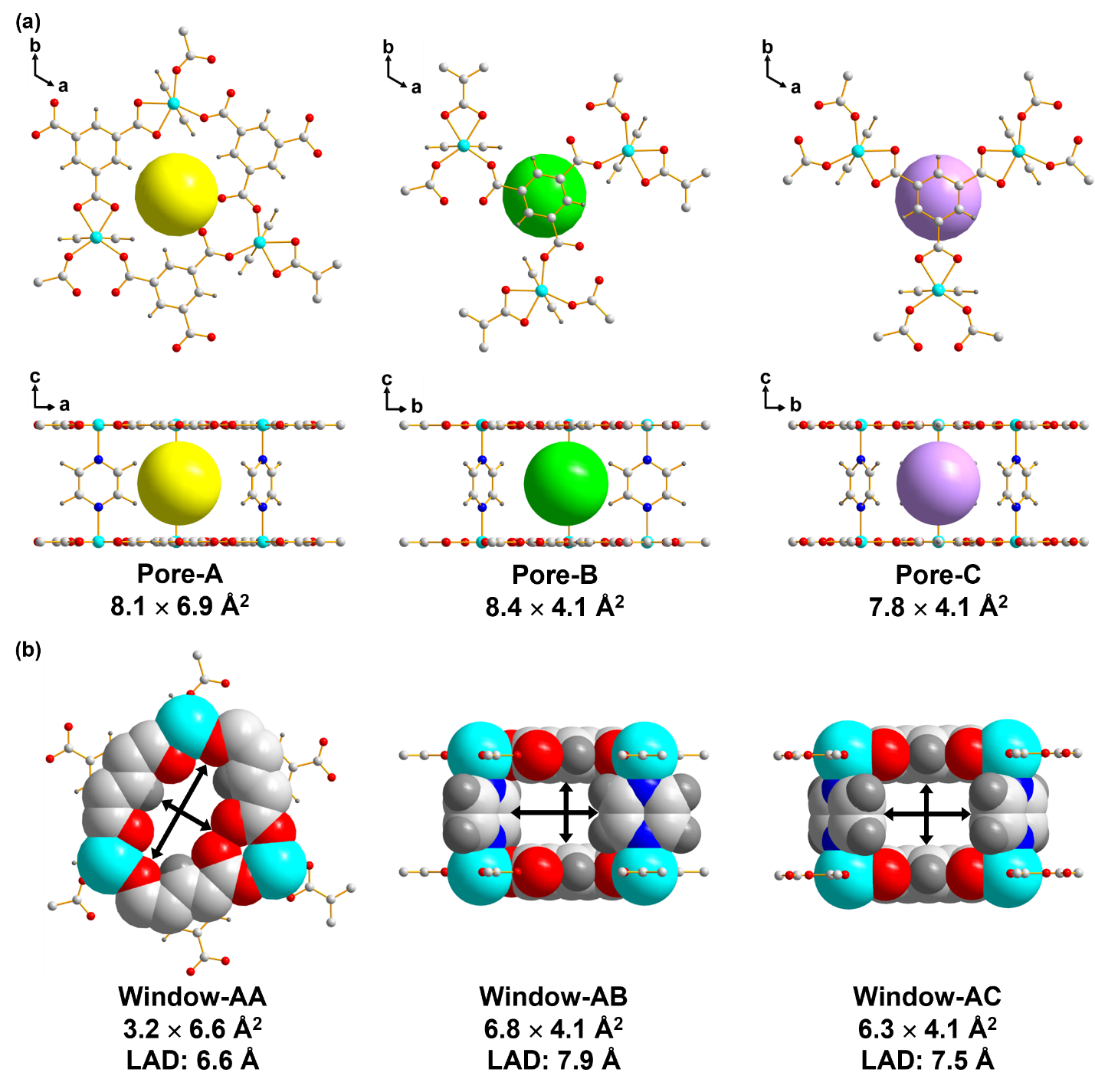


**Figure S3.** Pores and windows in Ni-HPZ. (a) Graphical representations of Pore-A, Pore-B, and Pore-C, shown in top view (top) and side view (bottom). (b) Graphical representations of individual windows. LAD was calculated as the largest diagonal distance within each window, considering pillar rotation.


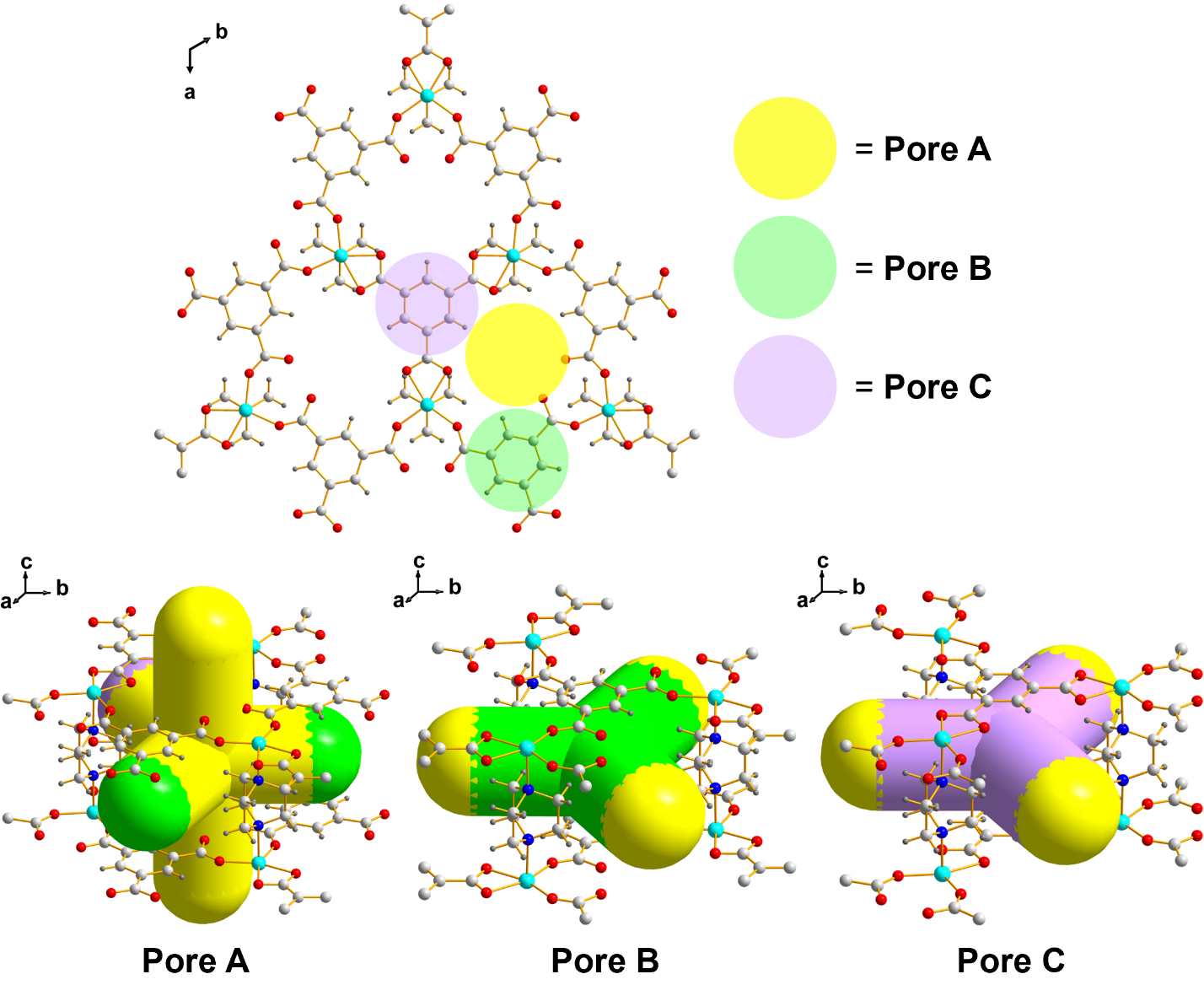


**Figure S4.** Three different types of pores and channels in the [Ni_3_(H_1.5_BTC)_2_(BTC)(Pillar)_3_] structure. The Ni-HDB structure was used for graphical representation. All pores and channels are visualized with a 2.5 Å radius, regardless of their actual dimensions.





**Figure S5.** Comparison of PXRD patterns of Ni-HDB under different conditions.


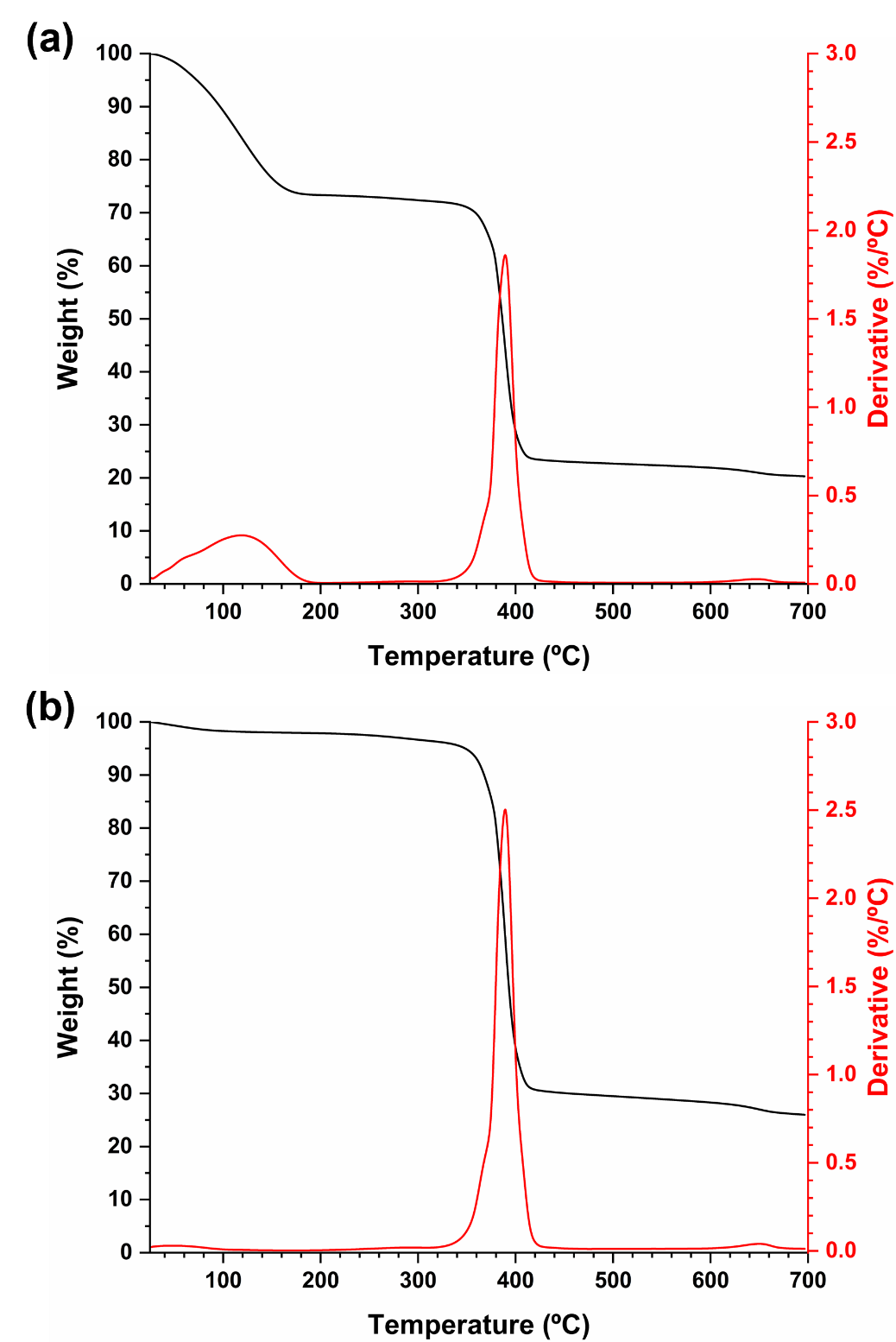


**Figure S6.** TGA of Ni-HDB under N_2_ flow. (a) Sample soaked in the volatile solvent DCM. (b) Sample activated at 120 °C under vacuum and then exposed to ambient air for 10 minutes.


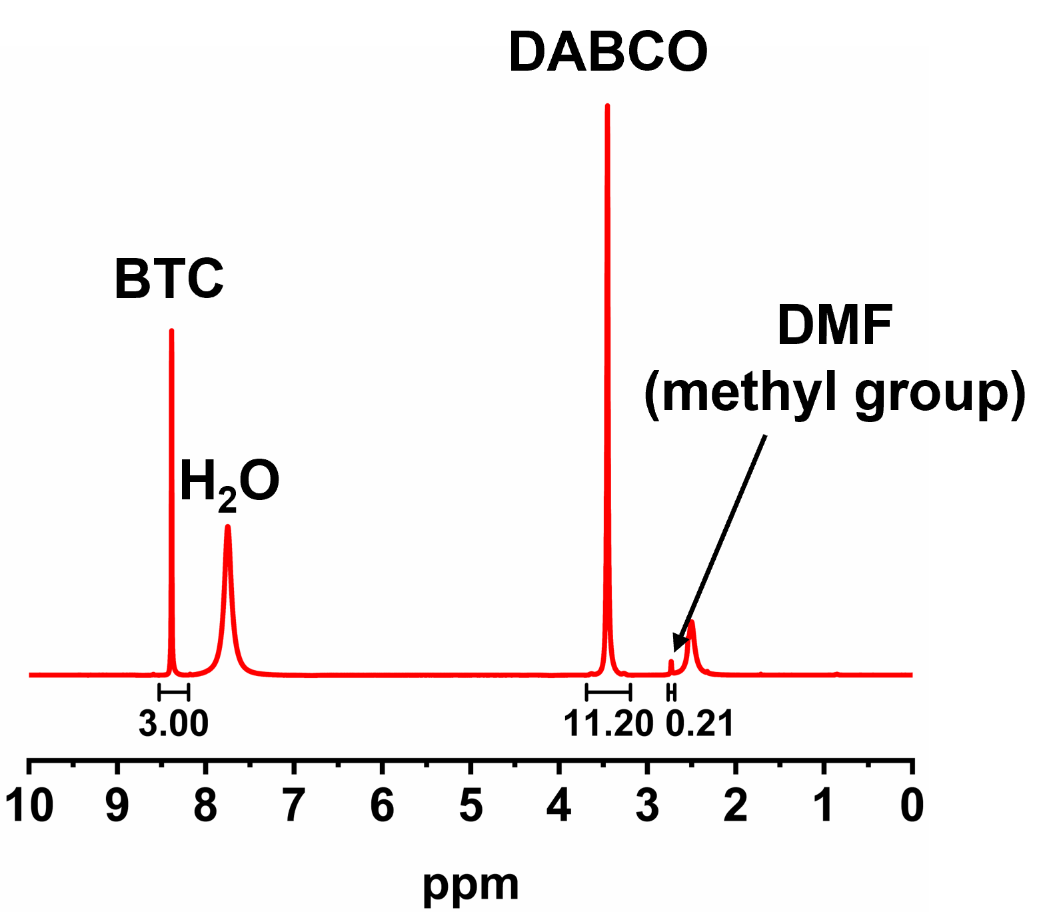


**Figure S7.** ^1^H NMR analysis of Ni-HDB activated at 120 °C under vacuum, digested in a DCl and DMSO-*d*_6_ mixed solution (DMSO-*d*_6_ peak at 2.5 ppm shown for reference).


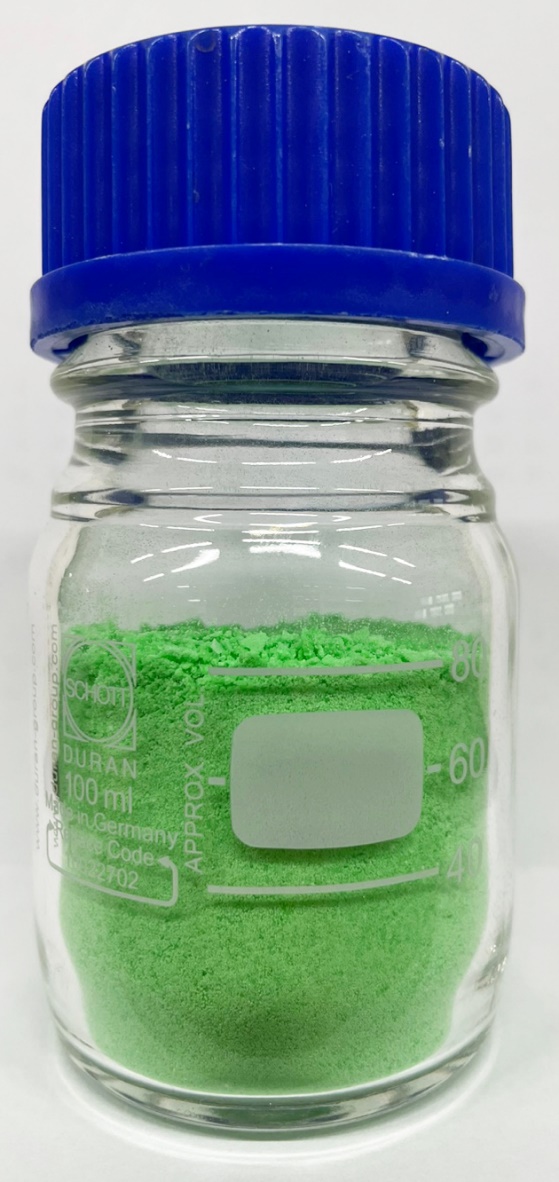


**Figure S8.** Photograph of bulk-scale synthesized Ni-HDB after activation.





**Figure S9.** Comparison of PXRD patterns of bulk-scale synthesized Ni-HDB under different conditions.


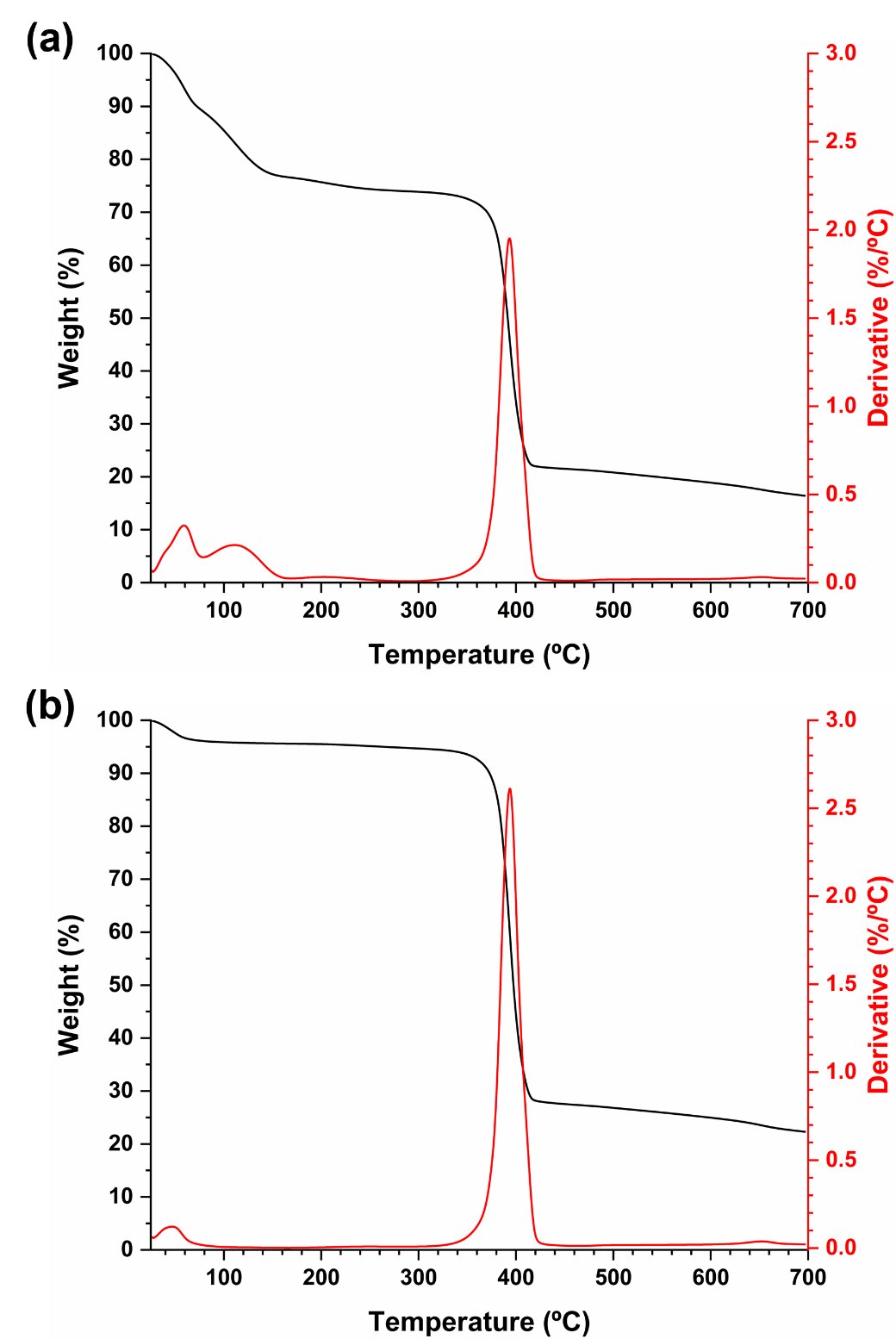


**Figure S10.** TGA of bulk-scale synthesized Ni-HDB under N_2_ flow. (a) Sample soaked in DCM. (b) Sample activated at 120 °C under vacuum and exposed to ambient air for 10 minutes.


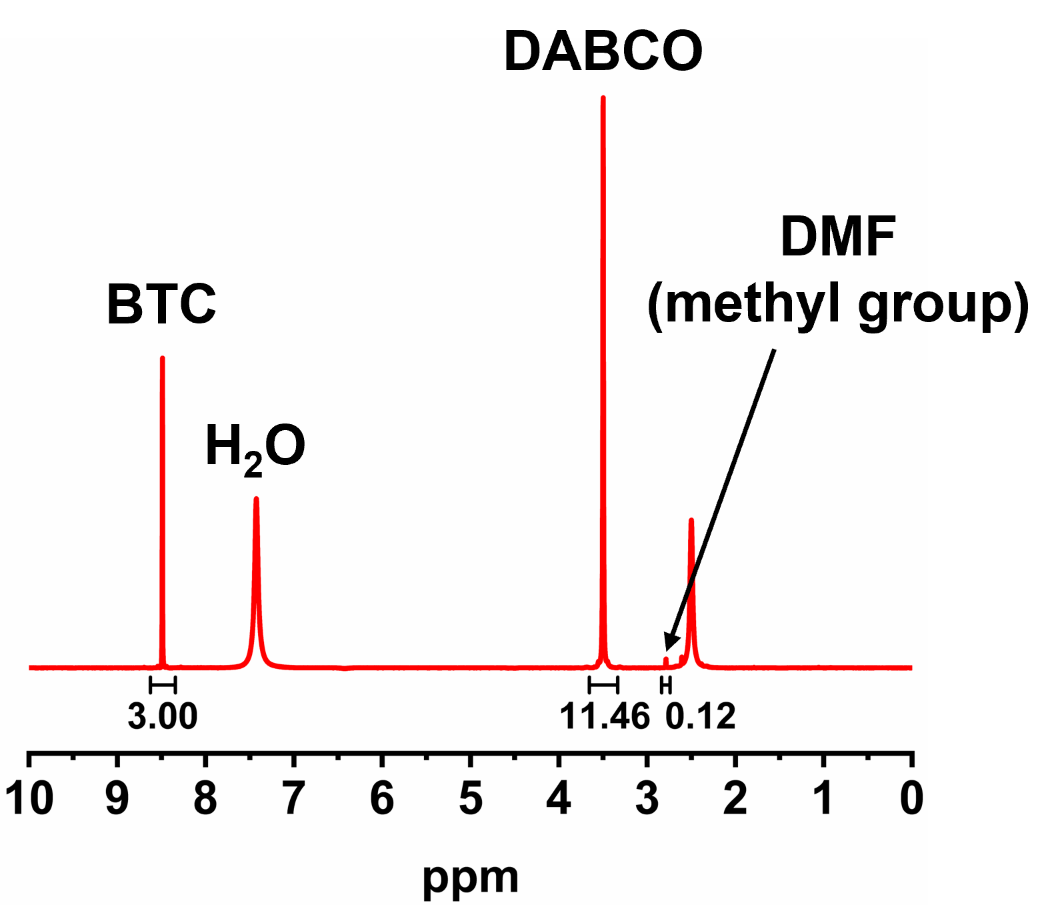


**Figure S11.** ^1^H NMR analysis of bulk-scale synthesized Ni-HDB activated at 120 °C under vacuum, digested in a DCl and DMSO-*d*_6_ mixed solution (DMSO-*d*_6_ peak at 2.5 ppm shown for reference).





**Figure S12.** N_2_ sorption experiments of Ni-HDB and bulk-scale synthesized Ni-HDB at 77 K. The bulk-scale sample exposed to ambient air for 2 months showed no decrease in porosity, indicating excellent air stability.





**Figure S13.** Pore size distribution of bulk-scale synthesized Ni-HDB, determined by nonlocal density functional theory (NLDFT).





**Figure S14.** PXRD patterns of bulk-scale synthesized Ni-HDB before and after exposure to ambient air for 2 months.





**Figure S15.** IAST adsorption selectivities of equimolar PX/OX, MX/OX, and PX/MX mixtures at 300 K in Ni-HDB.


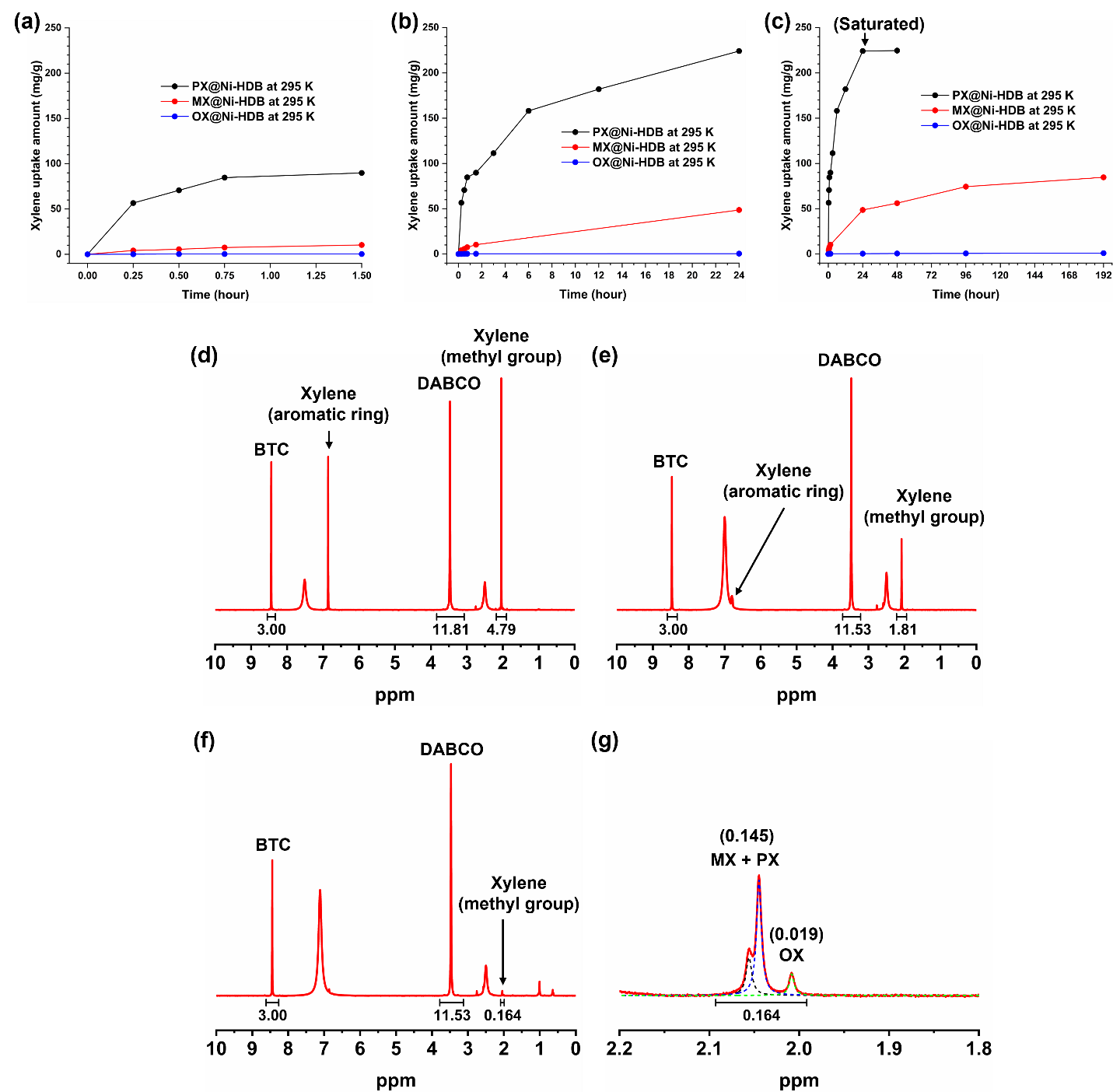


**Figure S16.** Adsorption kinetics of Ni-HDB at 295 K over various time periods: (a) 0–1.5 h, (b) 0–24 h, and (c) 0–192 h. ^1^H NMR analyses at saturation for (d) PX@Ni-HDB after 1 day, (e) MX@Ni-HDB after 8 days, and (f, g) OX@Ni-HDB after 8 days, performed by digesting the crystals in a DCl and DMSO-*d*_6_ solution. DMSO-*d*_6_ peak at 2.5 ppm is shown for reference. Uptake amounts were calculated based on the –CH_3_ peaks of xylene.


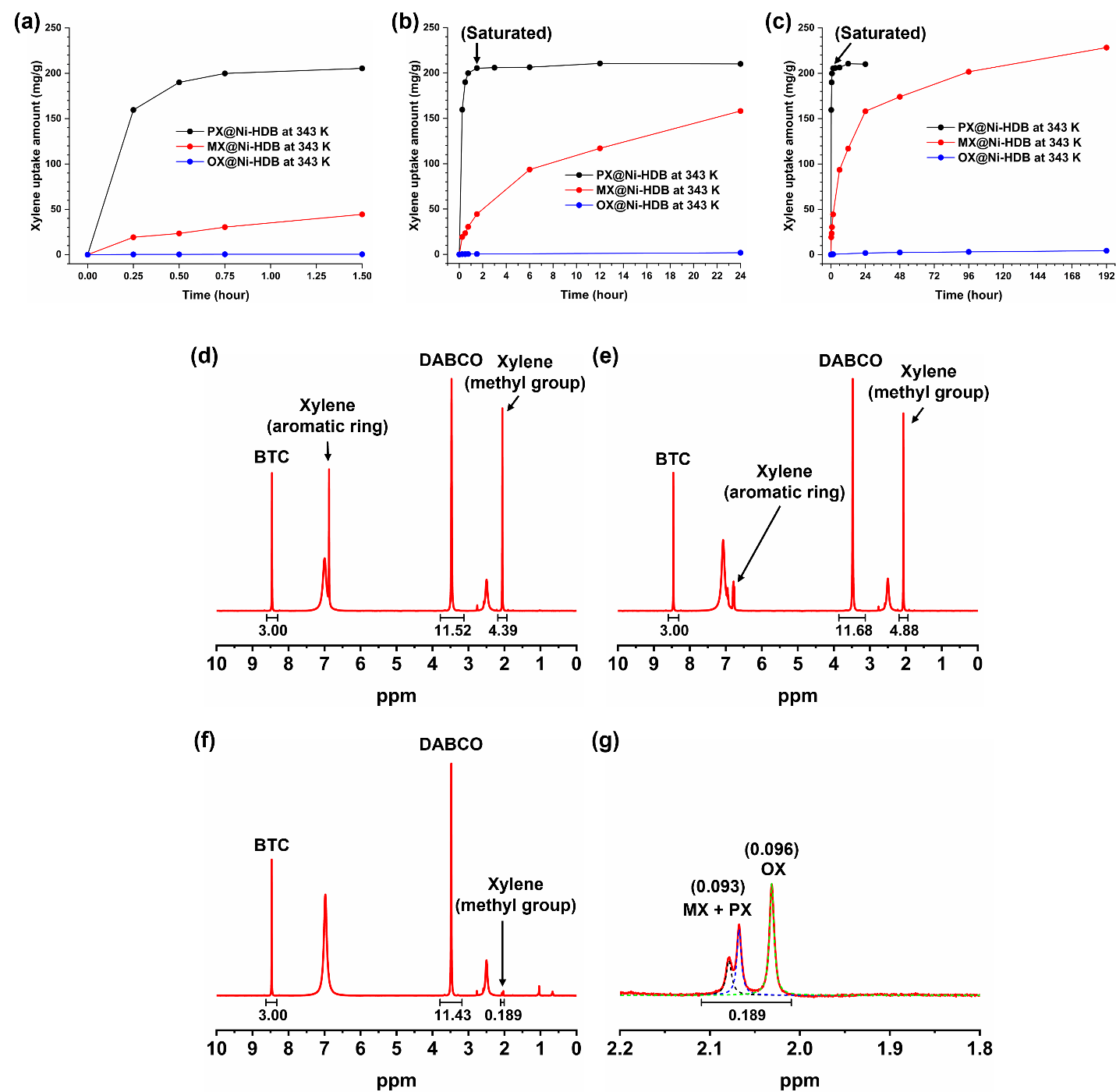


**Figure S17.** Adsorption kinetics of Ni-HDB at 343 K over various time periods: (a) 0–1.5 h, (b) 0–24 h, and (c) 0–192 h. ^1^H NMR analyses at saturation for (d) PX@Ni-HDB after 90 min, (e) MX@Ni-HDB after 8 days, and (f, g) OX@Ni-HDB after 8 days. Uptake amounts were calculated using –CH_3_ peaks of xylene.


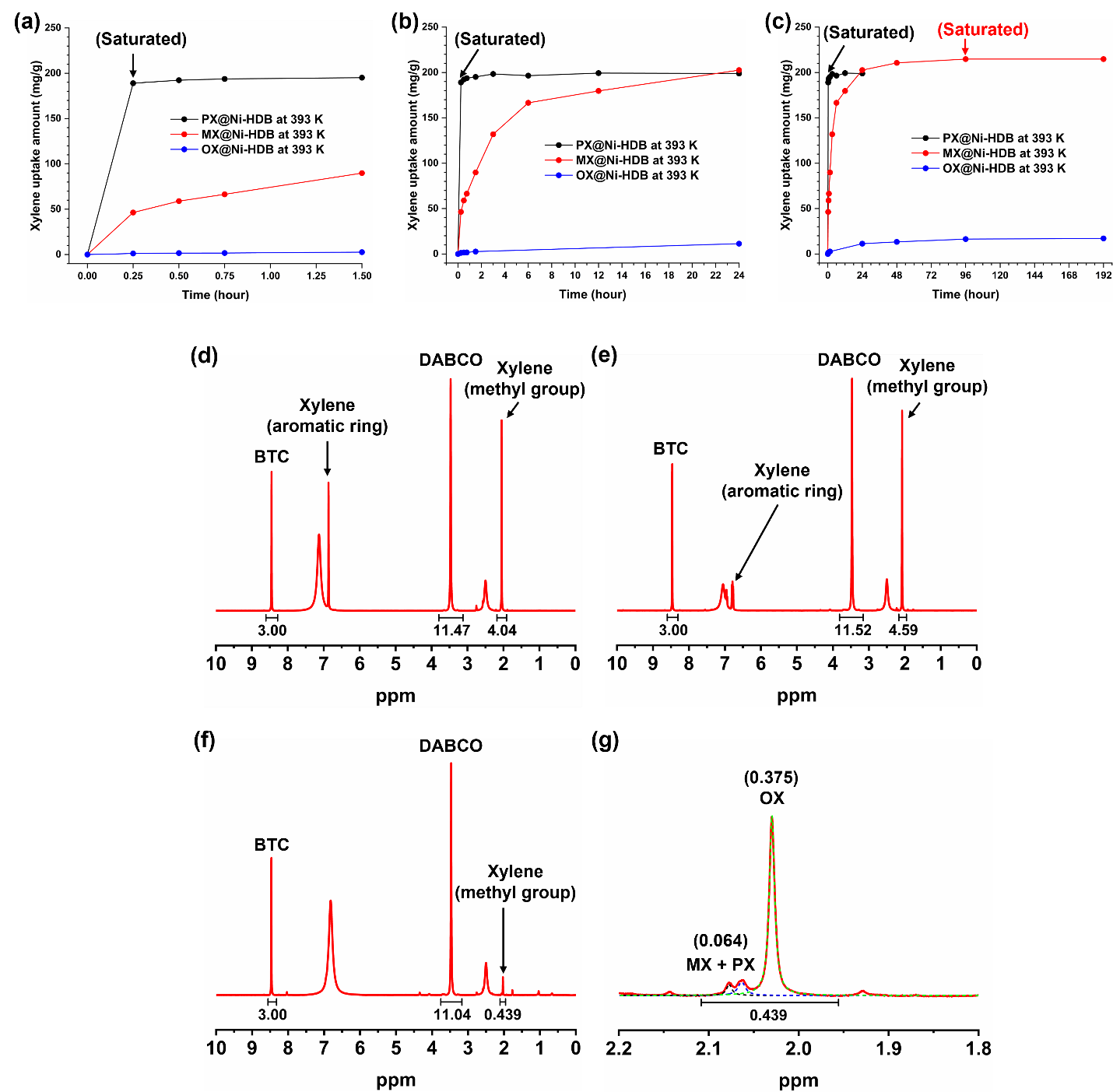


**Figure S18.** Adsorption kinetics of Ni-HDB at 393 K over various time periods: (a) 0–1.5 h, (b) 0–24 h, and (c) 0–192 h. ^1^H NMR analyses at saturation for (d) PX@Ni-HDB after 15 min, (e) MX@Ni-HDB after 4 days, and (f, g) OX@Ni-HDB after 8 days. Uptake amounts were determined from –CH_3_ peaks of xylene.


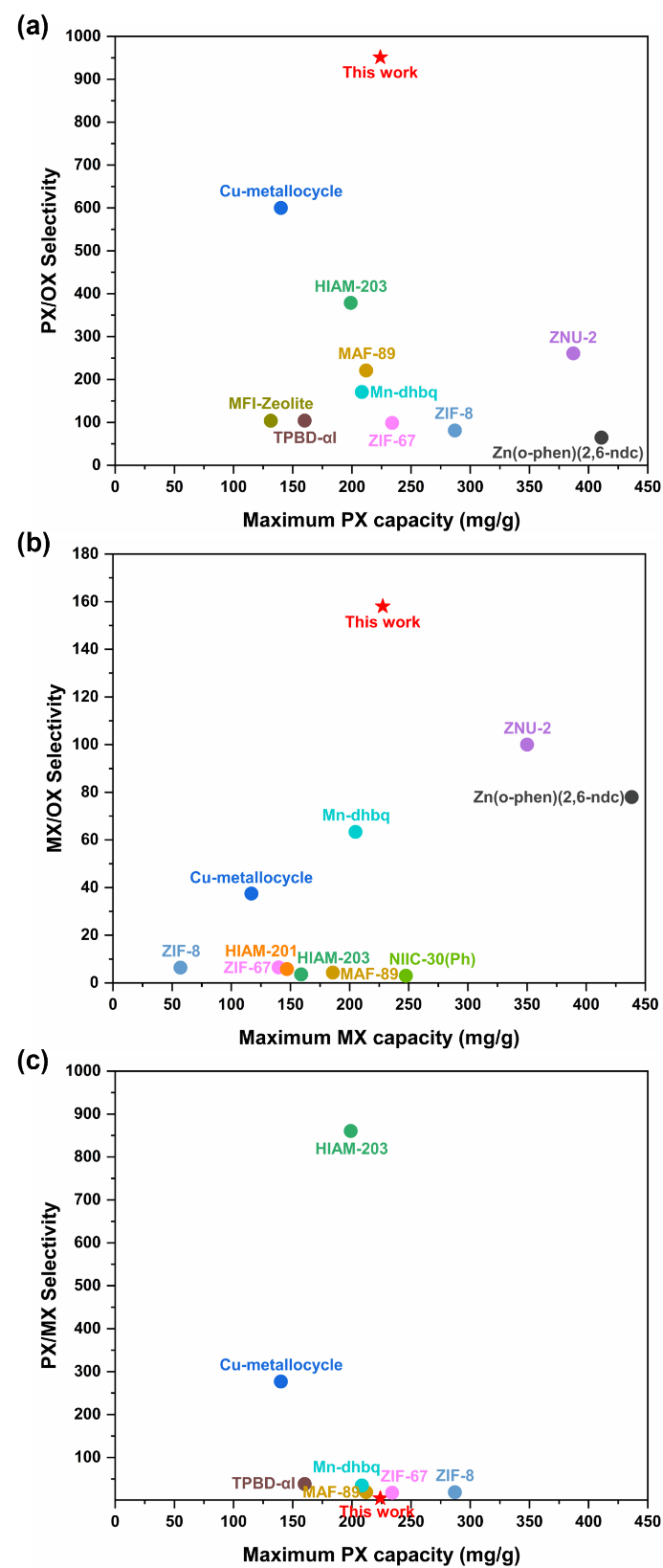


**Figure S19.** Comparison of adsorbents for separation of (a) PX/OX, (b) MX/OX, and (c) PX/MX. For each reported material, only the highest selectivity and capacity values were used.





**Figure S20.** GC results of liquid-phase single- and multi-component batch adsorption experiments after 1 day at 295 K. Peak positions of pure liquids are shown as references; all peak intensities were normalized.


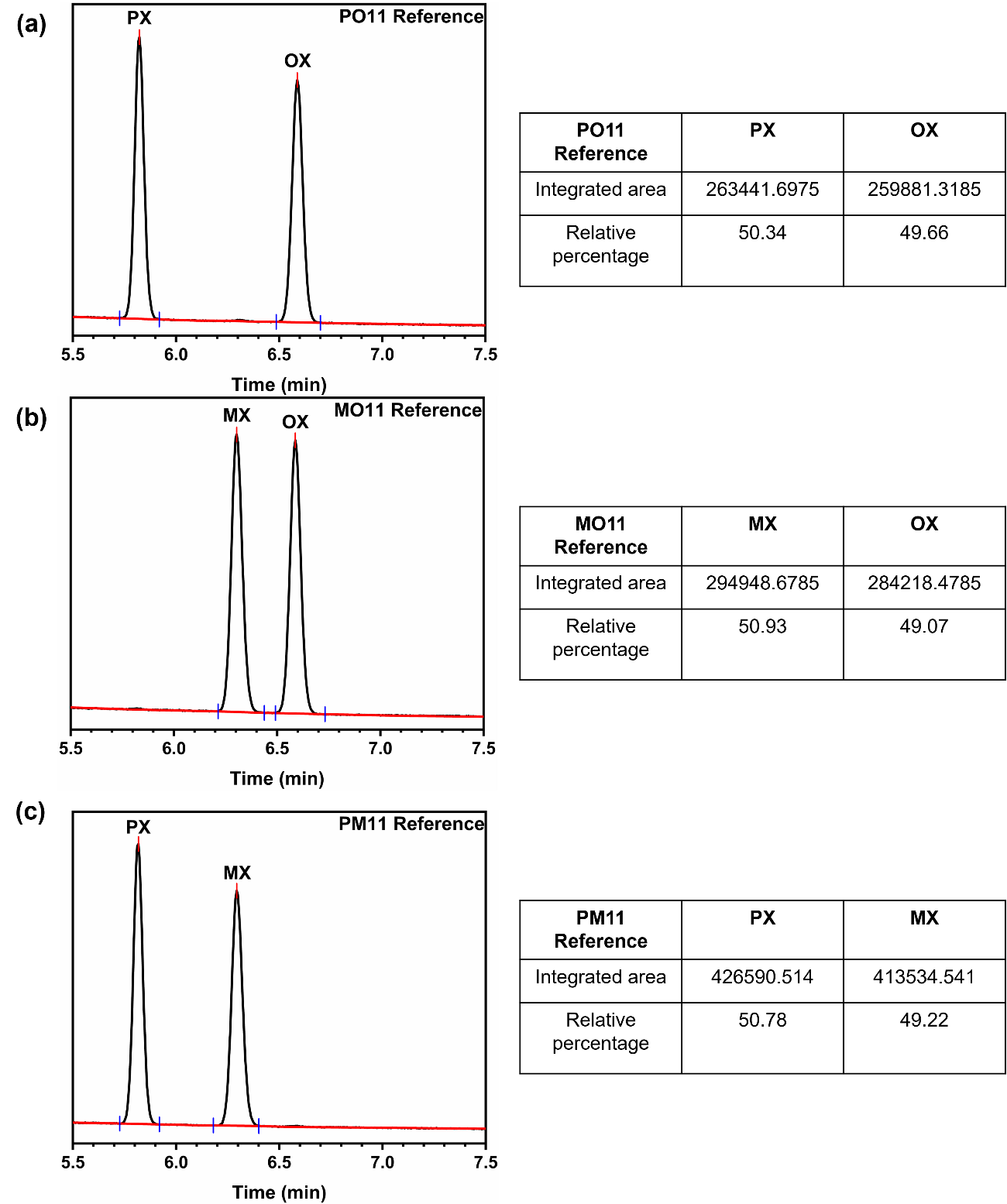


**Figure S21.** GC and peak integration results of binary (1:1) xylene solutions: (a) PX:OX = 1:1, (b) MX:OX = 1:1, and (c) PX:MX = 1:1. In-lab synthesized mixtures were used as calibration standards for accurate selectivity calculations.


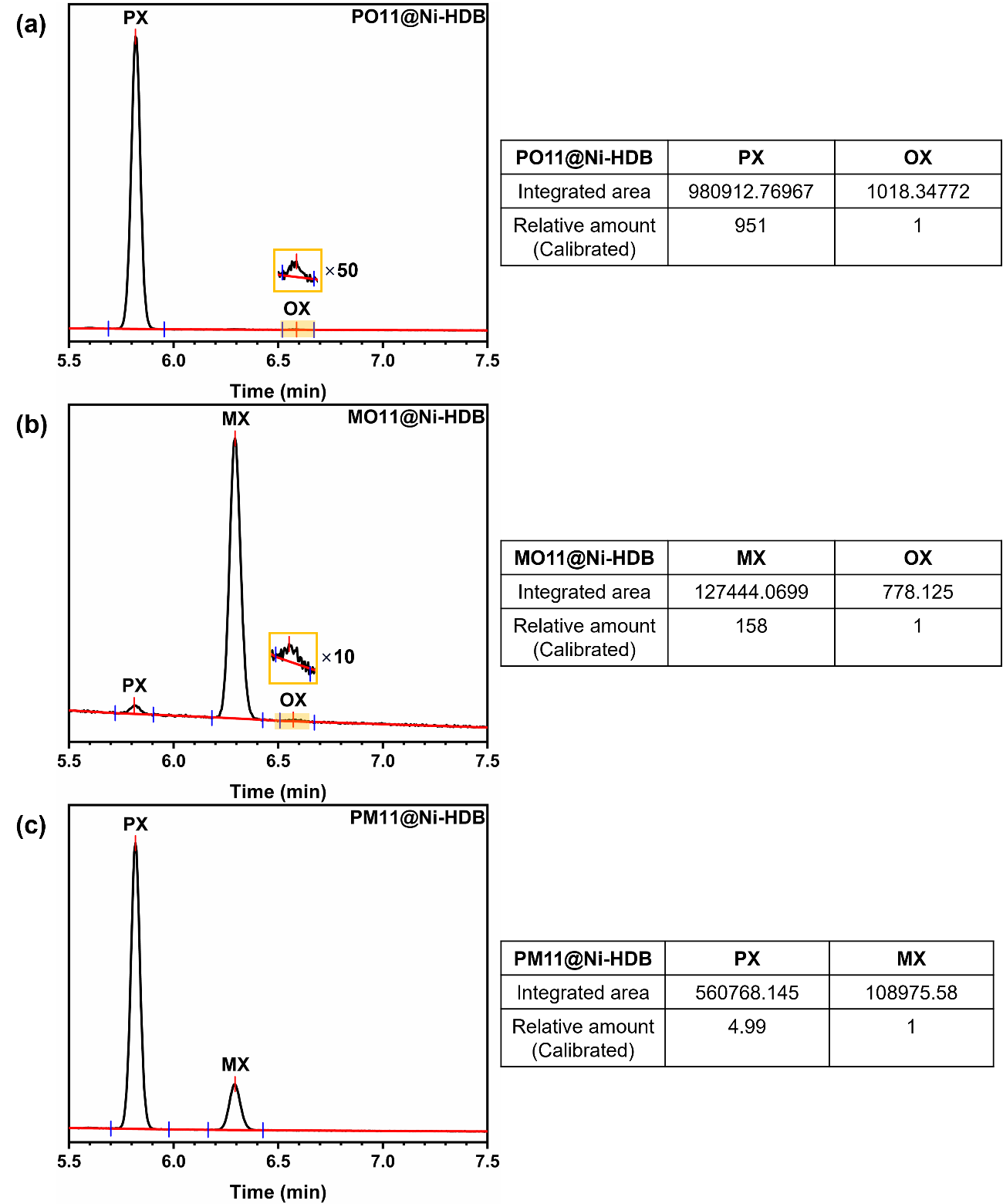


**Figure S22.** GC and peak integration results of Ni-HDB soaked in binary (1:1) xylene mixtures: (a) PX:OX = 1:1, (b) MX:OX = 1:1, and (c) PX:MX = 1:1.


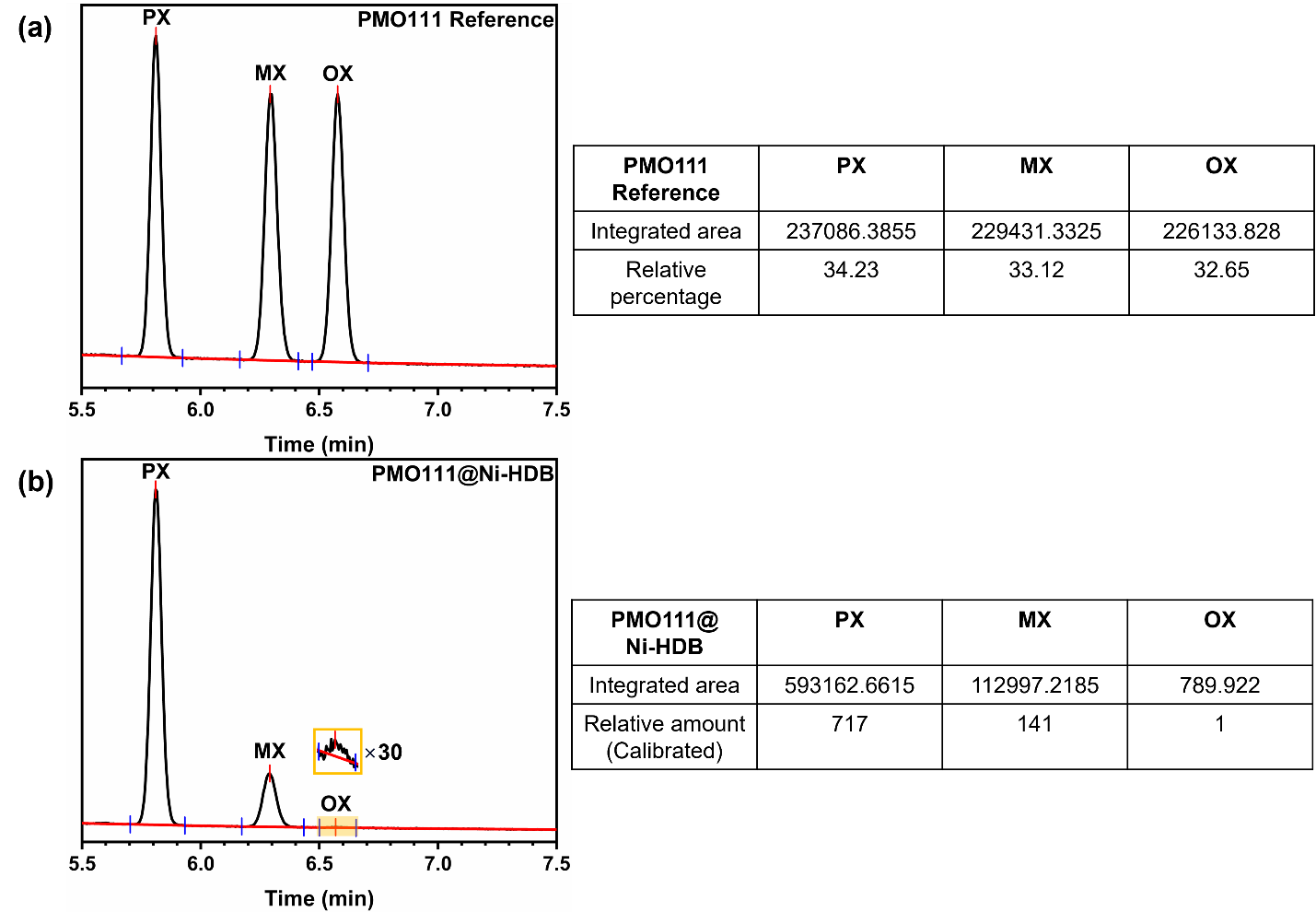


**Figure S23.** GC and peak integration results for (a) ternary (1:1:1) xylene solutions and (b) Ni-HDB soaked in these ternary mixtures. Calibration was performed using an in-lab synthesized 1:1:1 mixture to ensure accuracy in selectivity calculations.


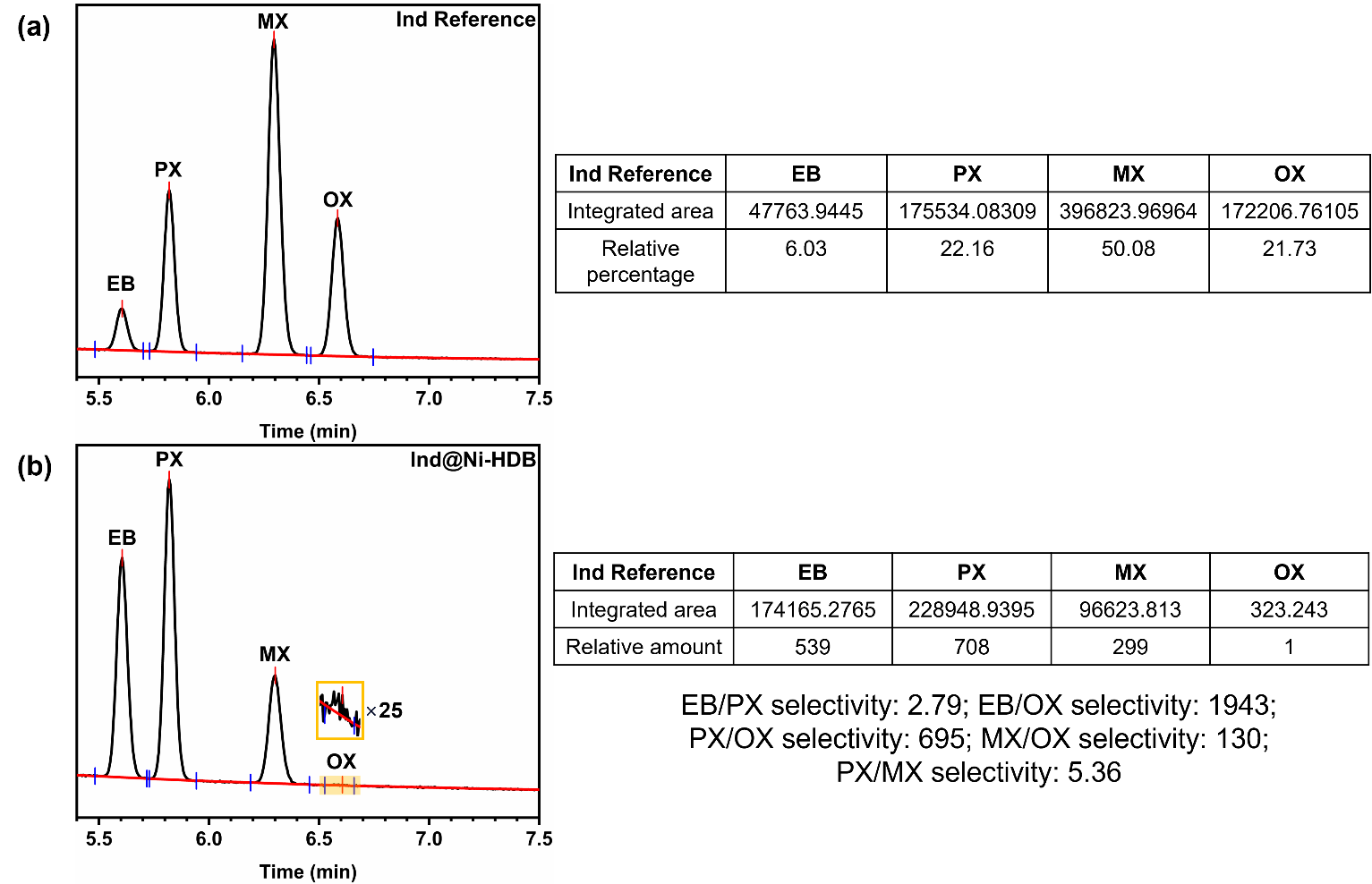


**Figure S24.** GC chromatograms and peak integration results for (a) the industrial xylene mixture (PX:MX:OX:EB = 22:50:22:6) and (b) the same mixture after adsorption by Ni-HDB. Calibration was performed using an in-house synthesized standard solution with the same industrial composition to ensure accuracy in selectivity calculations.


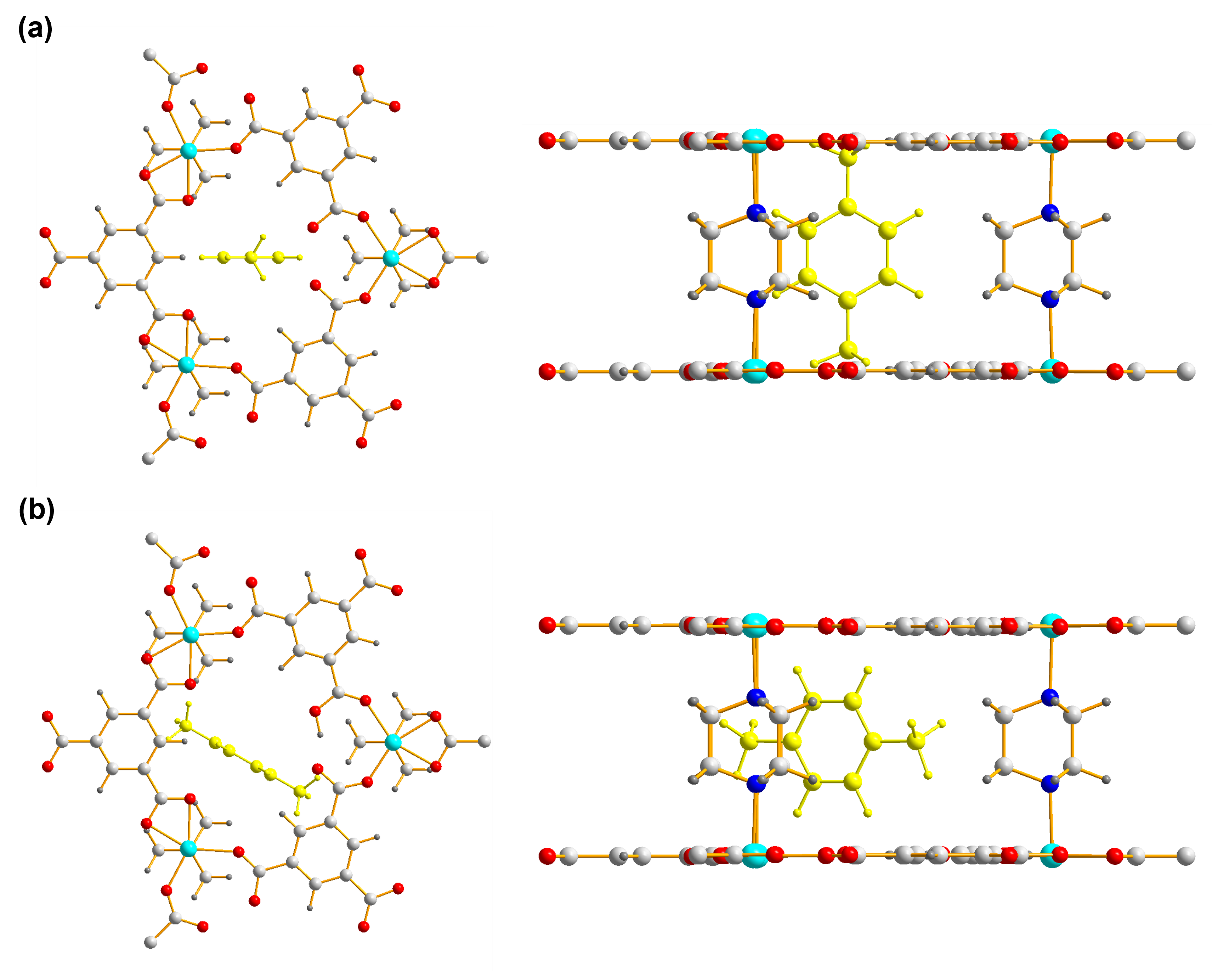


**Figure S25.** Two initial structures (a, b) for PX@Ni-HDB. Only the vertically oriented structure (a) converged during optimization, while the horizontally oriented structure (b) resulted in significant distortion.


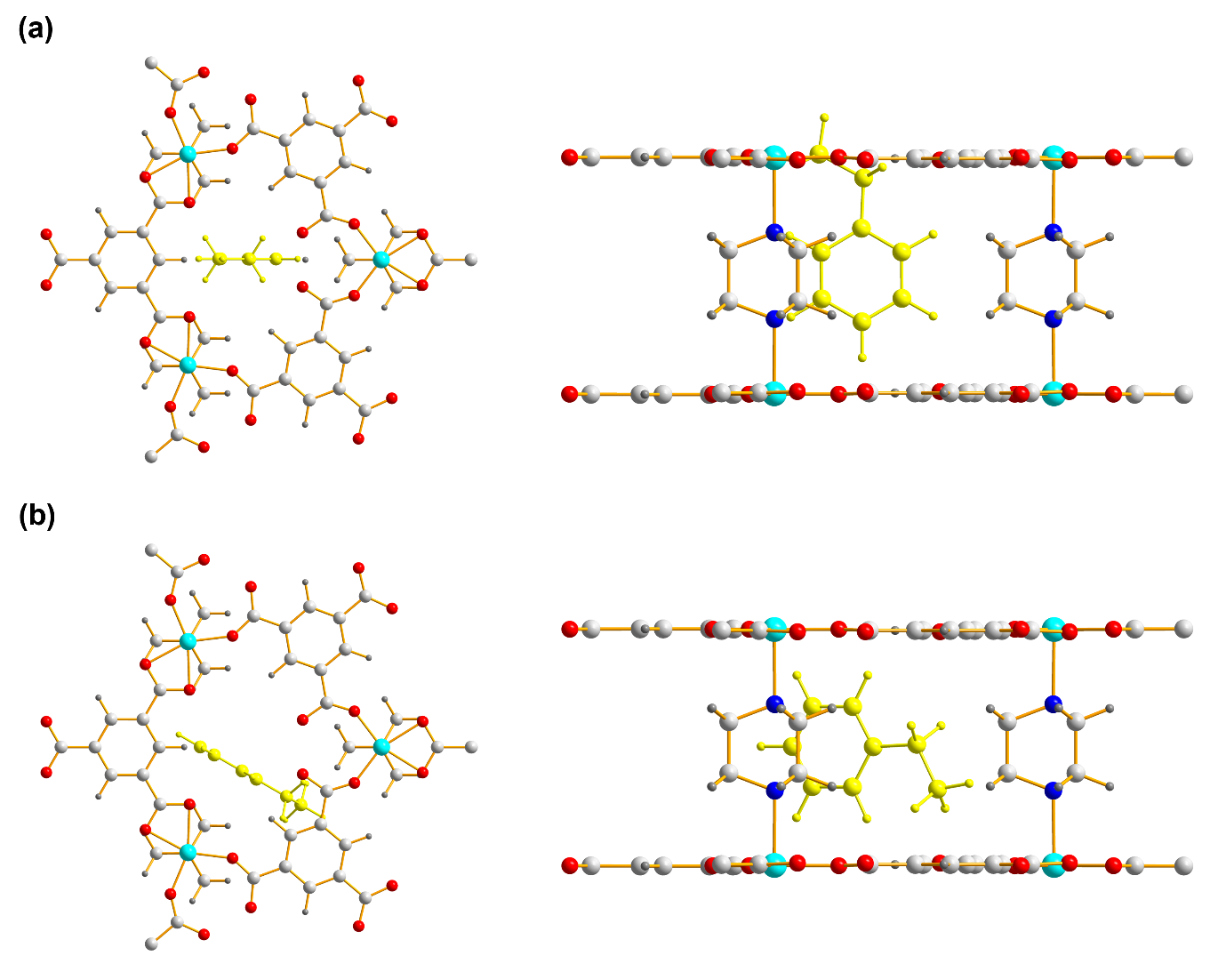


**Figure S26.** Two initial configurations of EB within Ni-HDB: (a) vertically oriented and (b) horizontally oriented. Only the vertical configuration (a) converged during geometry optimization, whereas the horizontal configuration (b) resulted in significant structural distortion.


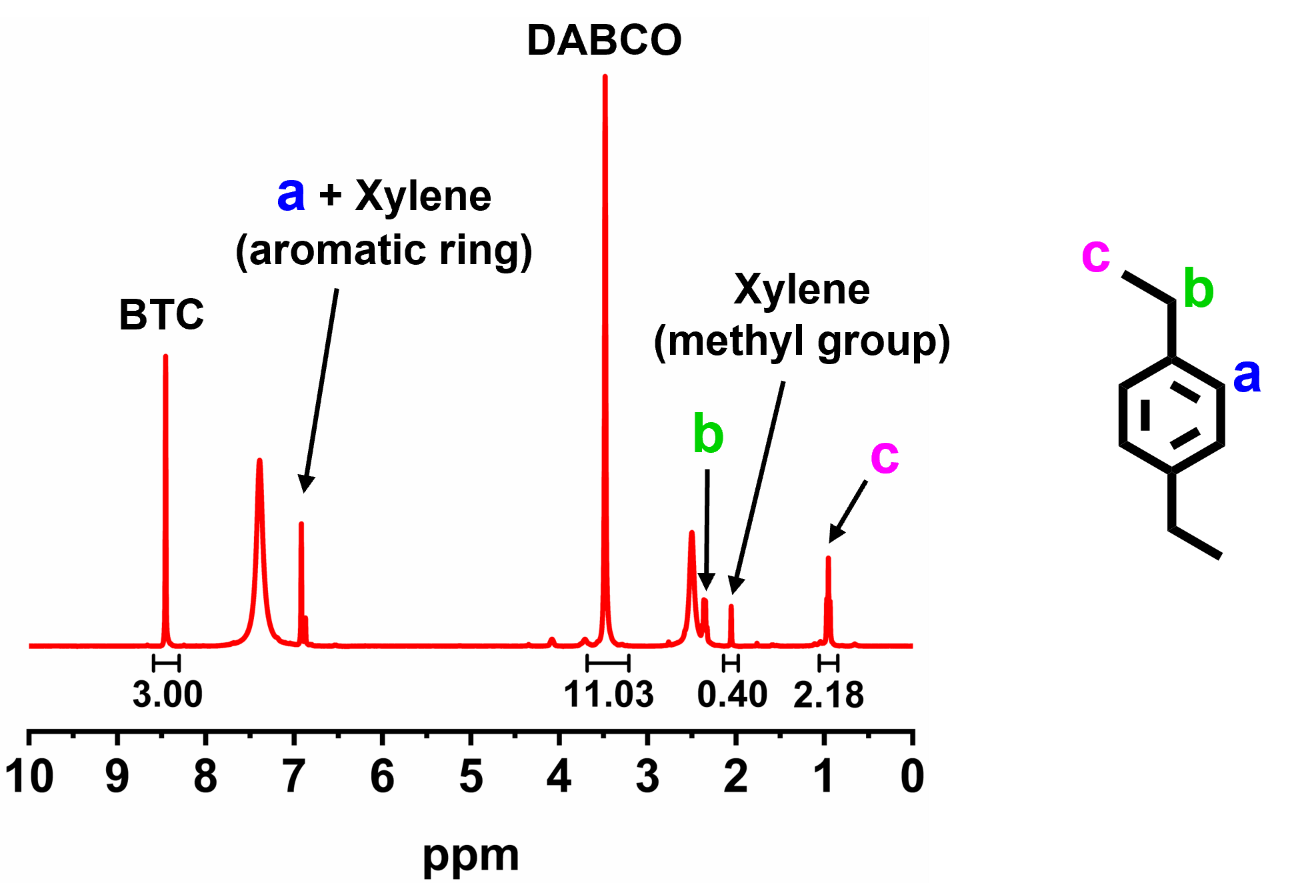


**Figure S27.** ^1^H NMR analysis of the PX@Ni-HDB sample after soaking in p-DEB solution at 120 °C for 1 day. The protons of p-DEB at different positions are labeled as a, b, and c. Uptake amounts were calculated based on the –CH_3_ peaks of PX and p-DEB.


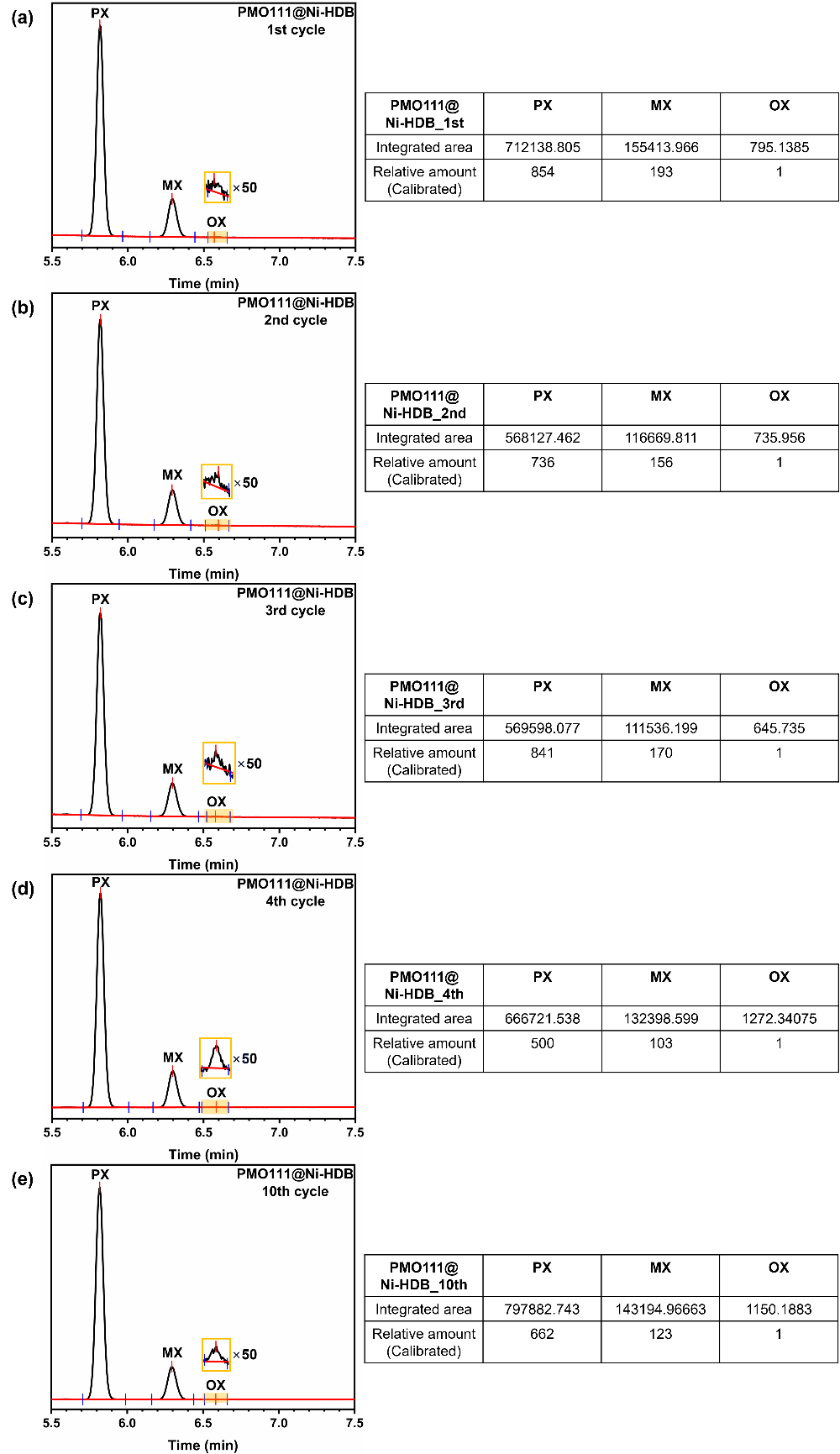


**Figure S28.** GC and peak integration results of Ni-HDB soaked in ternary (1:1:1) xylene mixtures after (a) 1st, (b) 2nd, (c) 3rd, (d) 4th, and (e) 10th adsorption cycle.


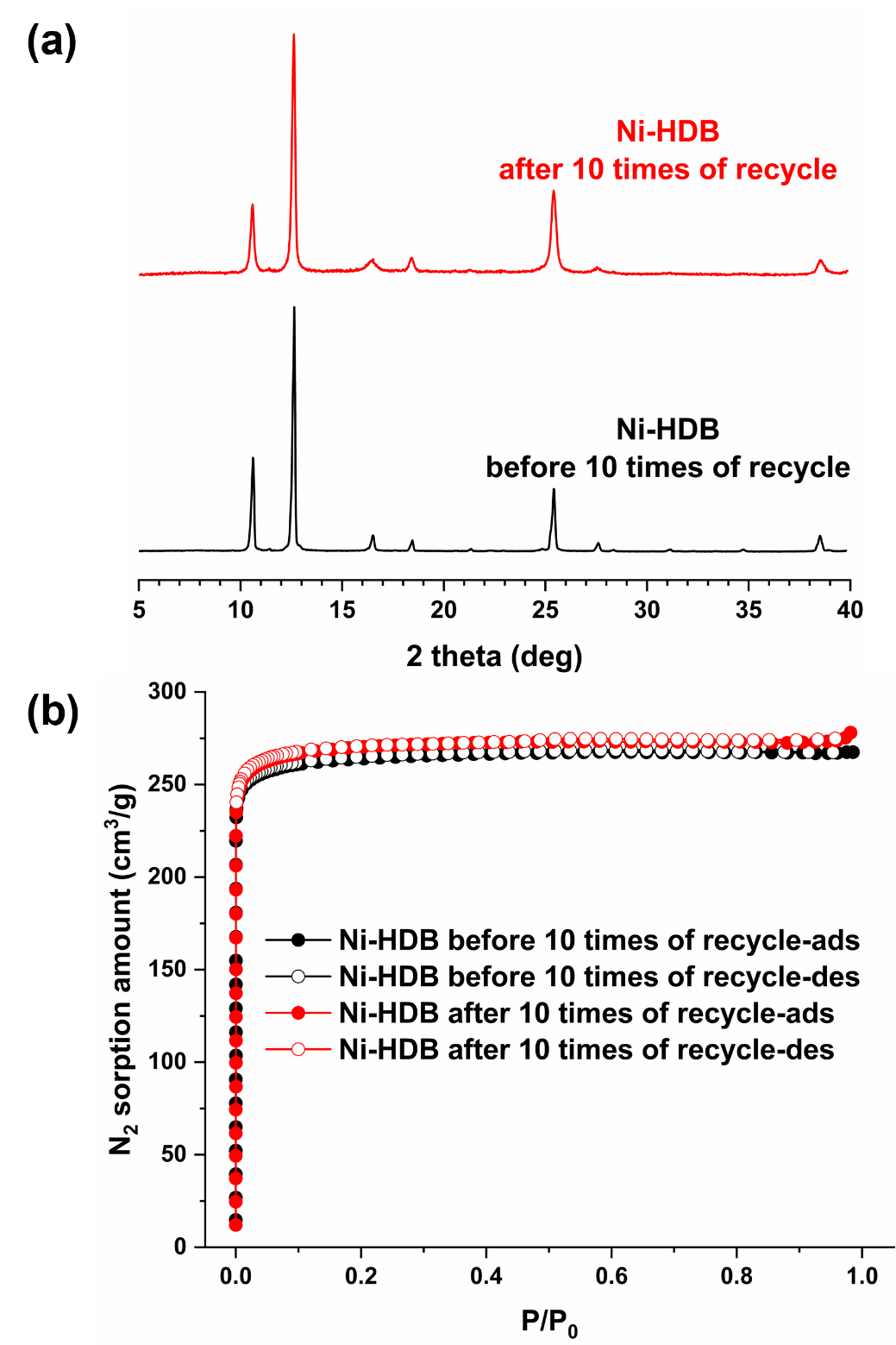


**Figure S29.** (a) PXRD pattern and (b) N_2_ sorption isotherms of Ni-HDB after 10 adsorption cycles, confirming the structural stability of Ni-HDB.


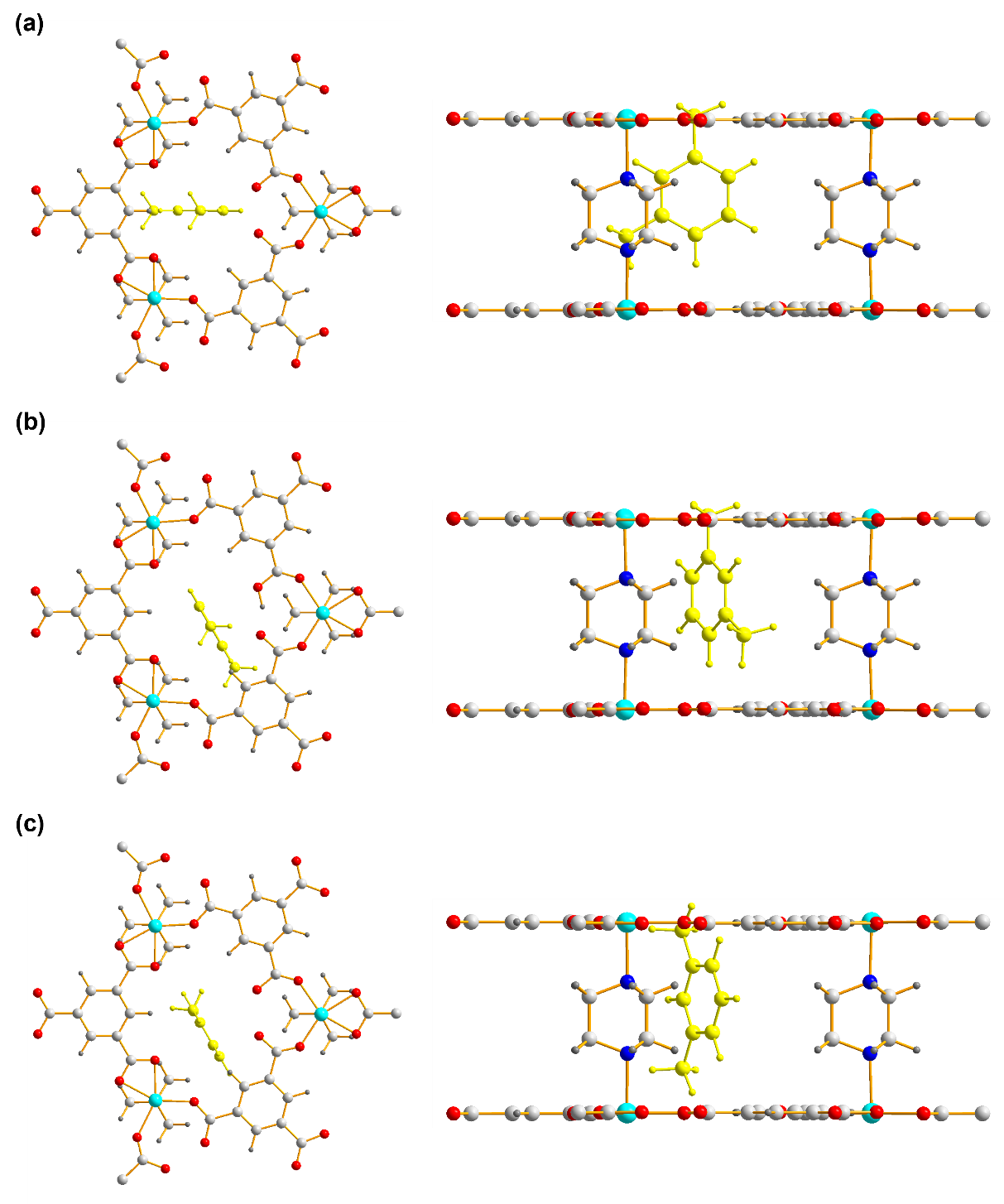


**Figure S30.** Three initial structures (a–c) for MX@Ni-HDB. Only structure (a) was successfully optimized. Structures (b) and (c) exhibited severe distortion due to steric interactions between methyl groups and the Ni-HDB framework.


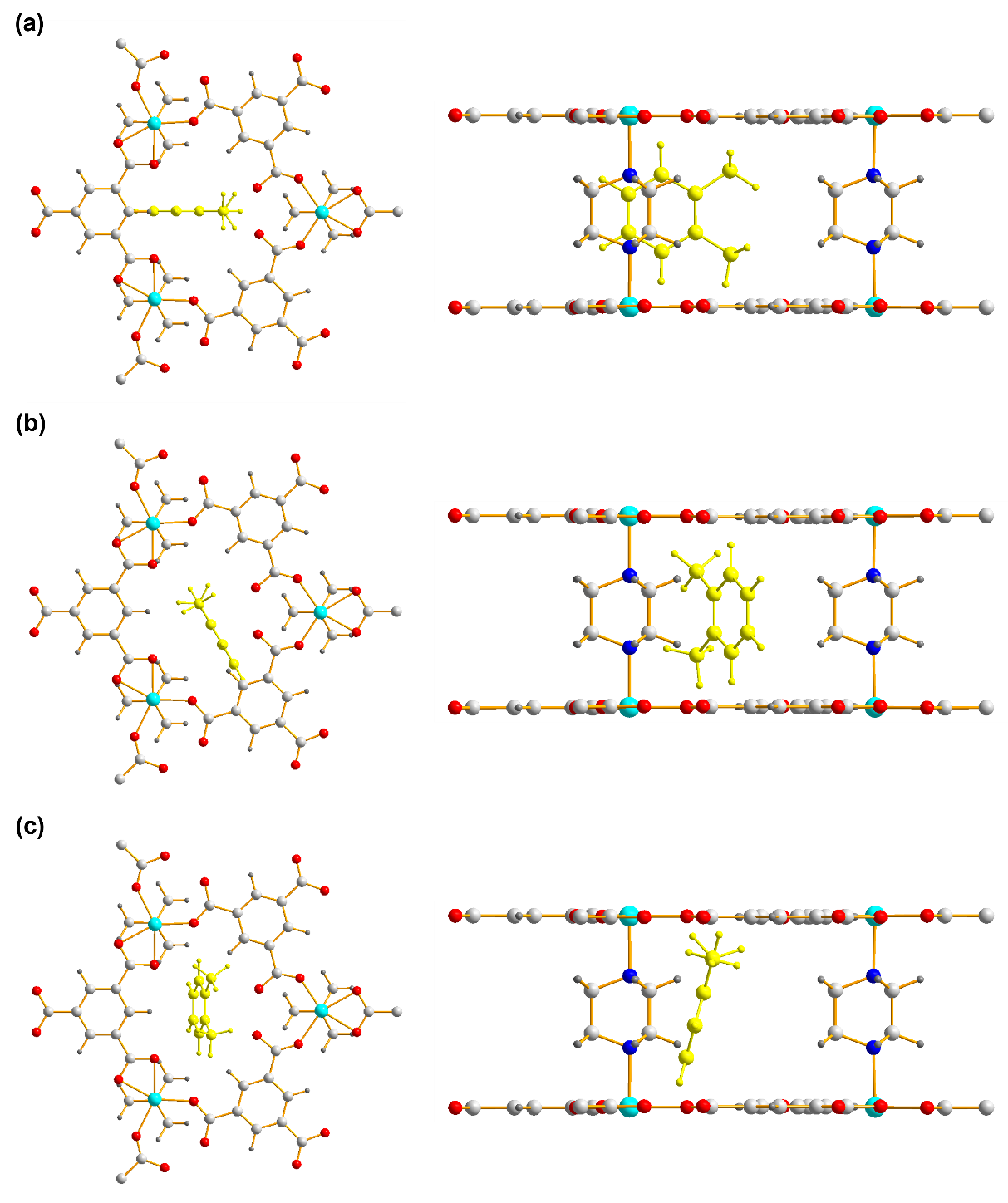


**Figure S31.** Three initial structures (a–c) for OX@Ni-HDB. Only structure (a) was successfully optimized. Structures (b) and (c) were highly distorted due to interactions between methyl groups in OX and the host framework.





**Figure S32.** PXRD patterns of Ni-HDB soaked in individual xylene isomers. The (–1 2 0) peak at 2θ = 10.608° and the (0 0 1) peak at 2θ = 12.633° shifted to lower angles, indicating unit cell expansion.





**Figure S33.** Simulated and experimental PXRD patterns of Ni-HBP and Ni-HPZ.


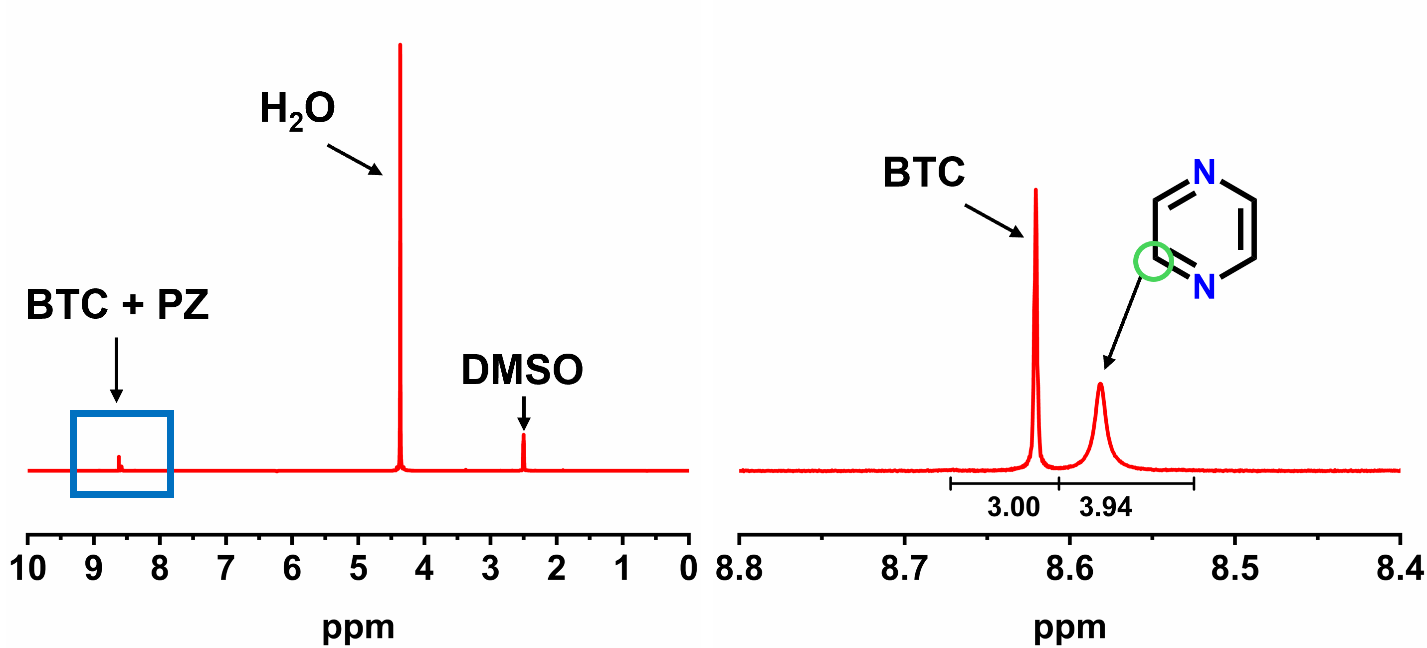


**Figure S34.** ^1^H NMR analysis of Ni-HPZ activated at 120 °C under vacuum, digested in a mixture of DCl (0.04 mL), DMSO-*d*_6_ (1 mL), and D_2_O (8 mL).


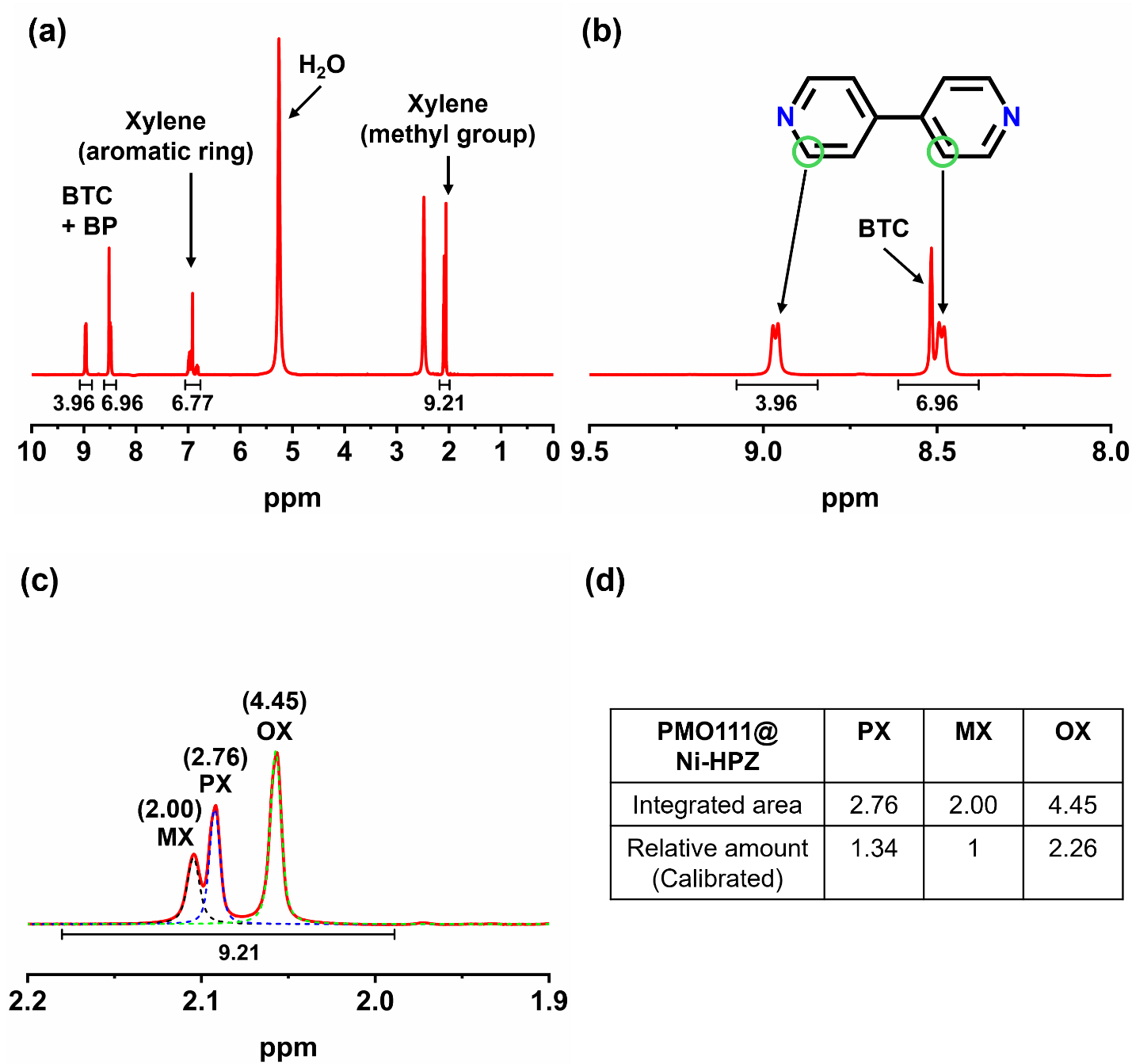


**Figure S35.** Liquid-phase batch adsorption results of Ni-HBP with a ternary (1:1:1) xylene isomer mixture at 295 K for 1 day. (a–c) ^1^H NMR analysis after digestion in a DCl and DMSO-*d*_6_ mixture (DMSO-*d*_6_ peak at 2.5 ppm for reference). (d) Calculated selectivities based on ^1^H NMR. Uptake was calculated using the –CH_3_ peaks of xylene.


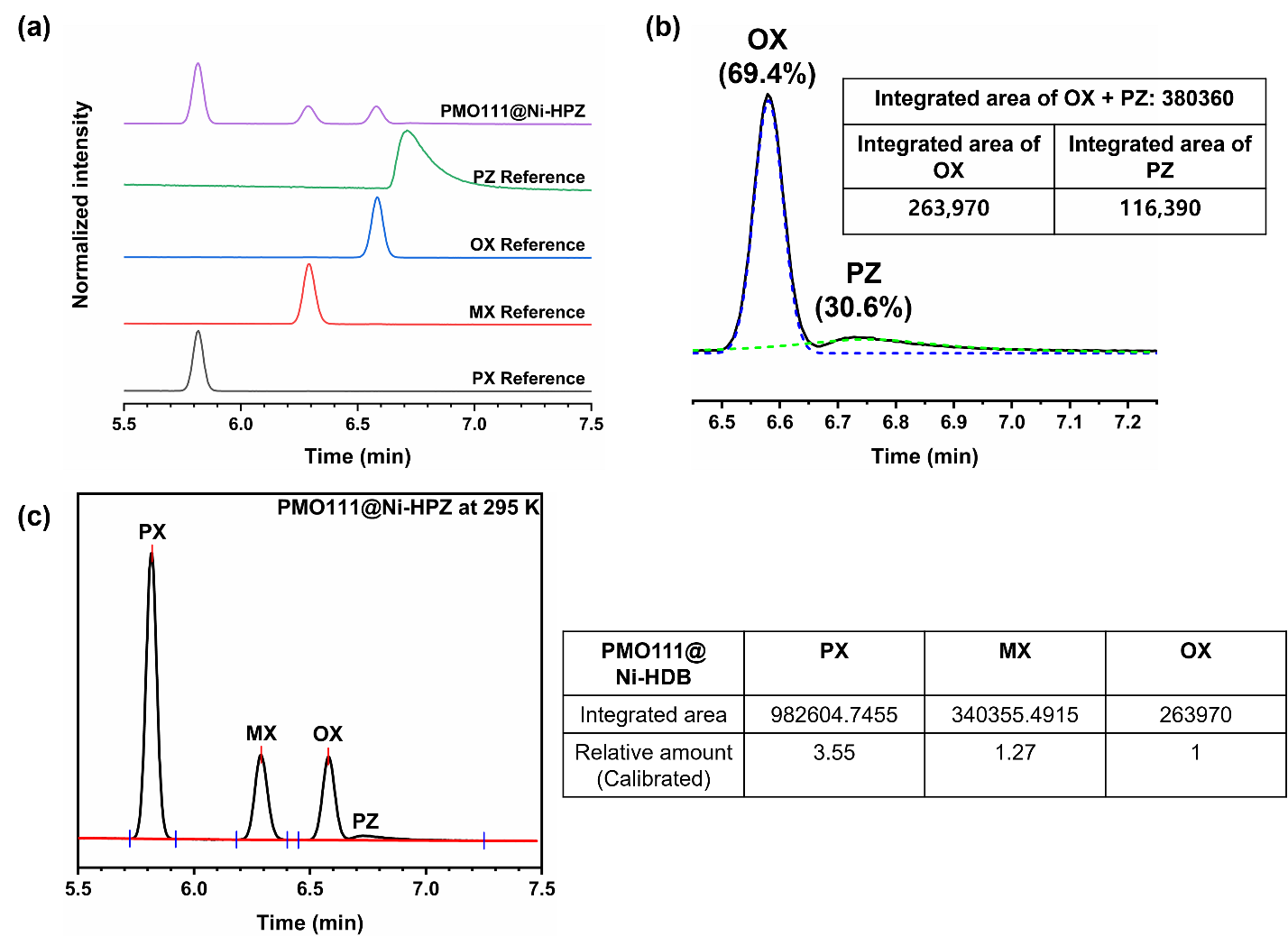


**Figure S36.** Liquid-phase batch adsorption results of Ni-HPZ with a ternary (1:1:1) xylene mixture at 295 K for 1 day. (a) GC analysis compared to references; PX, MX, and OX peaks were all detected in the Ni-HPZ sample. (b) Peak deconvolution of OX and PZ; a small amount of PZ dissolved into the CHCl_3_ phase was detected. Voigt fitting was applied. (c) GC peak integration results for PMO111@Ni-HPZ.

**Table S1.** Physical properties of xylene isomers.^[S15]­^ MIN-2 values of xylene isomers are obtained from crystallographic data.^[S16–S19]^

| Properties | PX | MX | OX | EB |
| --- | --- | --- | --- | --- |
| MIN-2, Å | 6.51 | 7.07 | 7.23 | 6.53 |
| Boiling Point, K | 411.5 | 412.3 | 417.6 | 409.3 |
| Melting Point, K | 286.4 | 222.5 | 248.0 | 178.2 |
| Density at 298 K, g/cm^3^ | 0.858 | 0.861 | 0.876 | 0.867 |
| Polarizability, cm^3^ | 13.7 | 14.2 | 14.9 | 14.2 |

**Table S2.** Crystal data and structure refinement parameters for Ni-HDB.

Empirical formula C_45_H_48_N_6_O_18_Ni_3_

Formula weight 1137.02

Temperature 100(2) K

Wavelength 0.700 Å

Crystal system Hexagonal

Space group *P*-62*m*

Unit cell dimensions a = 16.574(2) Å α = 90°

b = 16.574(2) Å β = 90°

c = 6.9370(14) Å γ = 120°

Volume 1650.3(6) Å^3^

Z 1

Density (calculated) 1.144 mg/m^3^

Absorption coefficient 0.867 mm^−1^

F(000) 588

Crystal size 0.090 × 0.085 × 0.010 mm^3^

Theta range for data collection 1.397 to 29.750°.

Index ranges -22<=h<=22, -23<=k<=23, -9<=l<=9

Reflections collected 17966

Independent reflections 1868 [R(int) = 0.1212]

Completeness to theta = 24.835° 99.5 %

Absorption correction Empirical

Max. and min. transmission 1.000 and 0.914

Refinement method Full-matrix least-squares on F^2^

Data / restraints / parameters 1868 / 0 / 75

Goodness-of-fit on F^2^ 1.030

Final R indices [I>2sigma(I)] R1 = 0.0917, wR2 = 0.2275

R indices (all data) R1 = 0.0920, wR2 = 0.2300

Absolute structure parameter 0.035(8)

Extinction coefficient 0.24(4)

Largest diff. peak and hole 2.993 and −2.406 e·Å^−3^

**Table S3.** Summary of reported adsorbents for the separation of PX/OX.

| **Adsorbent** | **Highest selectivity reported** | | **Composition** | **Refs** |
| --- | --- | --- | --- | --- |
|  | PX/OX | Condition |  |  |
| Ni-HDB | 951 | Liquid, 295 K | Binary (1:1) | This work |
|  | 717 | Liquid, 295 K | Ternary (1:1:1) | This work |
| Cu-metallocycle | 600 | Liquid, 303 K | Ternary (1:1:1) | [S18], [S19] |
| HIAM-203 | 378.8 | Vapor, 393 K  (IAST) | Ternary (1:1:1) | [S20], [S21] |
| JNU-2 | 261 | Vapor, 353 K  (Breakthrough) | Binary (1:1) | [S22] |
| MAF-89 | 221 | Liquid, 308 K | Binary (1:1) | [S23] |
| Mn-dhbq | 171 | Liquid, 393 K | Industrial^a^ | [S24] |
| TPBD-αI | 104.2 | Liquid, 298 K | Ternary (1:1:1) | [S20] |
| MFI-Zeolite | 104 | Liquid, 298 K | Binary (1:1) | [S25] |
| ZIF-67(Co) | 98.9 | Liquid, 298 K | Ternary (1:1:1) | [S26] |
| ZIF-8(Zn) | 81.2 | Liquid, 298 K | Ternary (1:1:1) | [S27] |
| Zn(o-phen)(2,6-ndc) | 64.6 | Liquid, 298 K | Binary (1:1) | [S27] |
| HIAM-201 | 24.25 | Vapor, 423 K | Ternary (1:1:1) | [S28] |
| AgLClO_4_ | 24 | Liquid, 383 K | Binary (1:1) | [S29] |
| NU-2000 | 20 | Liquid, 298 K | Ternary (1:1:1) | [S30] |
| sql-4,5-Zn | 19.01 | Liquid, 298 K | Ternary (1:1:1) | [S31] |
| AZO-Cage | 15.6 | Vapor, 298 K | Ternary (1:1:1) | [S32] |
| BAX | 2.82 | Liquid, 423 K  (Breakthrough) | Binary (1:1) | [S33] |
| KX | 2.43 | Liquid, 423 K  (Breakthrough) | Binary (1:1) | [S34] |
| MIL-125(Ti)-NH_2_ | 2.2 | Liquid, 298 K  (Breakthrough) | Binary (1:1) | [S35] |
| Zn(purine)I | N.A.^b^ | Liquid, 298 K | Ternary (1:1:1) | [S36] |
|  |  |  |  | [S37] |

a: Industrial composition of PX:MX:OX:Ethylbenzene = 22:50:22:6.

b: Negligible OX uptake was observed.

**Table S4.** Summary of reported adsorbents for the separation of MX/OX.

| **Adsorbent** | **Highest selectivity reported** | | **Composition** | **Refs** |
| --- | --- | --- | --- | --- |
|  | MX/OX | Condition |  |  |
| Ni-HDB | 158 | Liquid, 295 K | Binary (1:1) | This work |
|  | 141 | Liquid, 295 K | Ternary (1:1:1) | This work |
| JNU-2 | 100 | Vapor, 353 K  (Breakthrough) | Binary (1:1) | [S23] |
| Zn(o-phen)(2,6-ndc) | 78 | Liquid, 298 K | Binary (1:1) | [S28] |
| Mn-dhbq | 63.4 | Liquid, 393 K | Binary (1:1) | [S20] |
| Cu-metallocycle | 37.5 | Liquid, 295 K | Binary (1:1) | [S20], [S21] |
| ZIF-67(Co) | 6.5 | Liquid, 333 K | Ternary (1:1:1) | [S27] |
| ZIF-8(Zn) | 6.4 | Liquid, 363 K | Ternary (1:1:1) | [S27] |
| HIAM-201 | 5.8 | Vapor, 423 K | Ternary (1:1:1) | [S29] |
| MAF-89 | 4.33 | Liquid, 308 K | Ternary (1:1:1) | [S24] |
| sql-4,5-Zn | 4.23 | Liquid, 298 K | Binary (1:1) | [S32] |
| AgLClO_4_ | 3.93 | Liquid, 383 K | Binary (1:1) | [S30] |
| HIAM-203 | 3.56 | Vapor, 393 K | Ternary (1:1:1) | [S22] |
| NIIC-30(Ph) | 3.03 | Liquid, 298 K | Binary (1:1) | [S38] |
| TPBD-αI | 2.7 | Liquid, 298 K | Ternary (1:1:1) | [S25] |
| MFM-300(In) | 2.7 | Liquid, 293 K  (Breakthrough) | Ternary (1:1:1) | [S39] |

**Table S5.** Optimized unit cell parameters, cell volume changes, and total energies for Ni-HDB, PX@Ni-HDB, MX@Ni-HDB, and OX@Ni-HDB.

|  | **Ni-HDB** | **PX@Ni-HDB** | **MX@Ni-HDB** | **OX@Ni-HDB** | **EB@Ni-HDB** |
| --- | --- | --- | --- | --- | --- |
| ***a ,b* (Å)** | 16.39 | 16.45 | 16.40 | 16.34 | 16.40 |
| ***c* (Å)** | 7.07 | 7.50 | 7.36 | 7.52 | 7.61 |
| **V(abc)** | 1645.94 | 1756.93 | 1714.36 | 1738.41 | 1772.13 |
| **ΔV(abc) (%)** |  | 6.74 | 4.16 | 5.62 | 7.67 |
| ***E*_Xylene@Ni-HDB_ (kJ/mol)** | −156771.2 | −188717.5 | −188723.5 | −188688.8 | −188687.2 |
| ***E*_Xylene_ (kJ/mol)** |  | −10589.34 | −10589.55 | −10585.62 | −10575.21 |
| **B.E. (kJ/mol)** | **-** | −59.40 | −61.22 | −53.56 | −63.46 |

# References

[S1] Materials Studio, version 4.3; Accelrys: San Diego, CA, **2008**.

[S2] J. Seong, S. Jeong, S. W. Moon, S. Lee, J. Lim, A. Sharma, S. Won, S. B. Baek, S. K. Min, M. S. Lah, “Multivariate Metal−Organic Frameworks Ranging from a Homogeneous Uniform Distribution to Heterogeneous 1D, 2D, and 3D Distributions of Mixed Building Blocks” *Chem. Mater.* **2024**, *36*, 925–936.

[S3] S. Jeong, D. Kim, S. Shin, D. Moon, S. J. Cho, M. S. Lah, “Combinational Synthetic Approaches for Isoreticular and Polymorphic Metal–Organic Frameworks with Tuned Pore Geometries and Surface Properties” *Chem. Mater.* **2014**, *26*, 1711–1719.

[S4] PAL BL2D-SMDC Program: J. W. Shin, K. Eom, D. Moon, “BL2D-SMC, the supramolecular crystallography beamline at the Pohang Light Source II, Korea” *J. Synchrotron Rad*. **2016**, *23*, 369–373.

[S5] Z. Otwinowski, W. Minor, “Processing of X-ray diffraction data collected in oscillation mode” *Methods in Enzymology*, **1997**, *276*, 307–326.

[S6] SHELX Program: G. M. Sheldrick, “Crystal structure refinement with SHELXL” *Acta Crystallogr., Sect. C: Struct. Chem*. **2015**, *C71*, 3–8.

[S7] PLATON Program: A. L. Spek, “PLATON SQUEEZE: a Tool for the Calculation of the Disordered Solvent Contribution to the Calculated Structure Factors” *Acta Crystallogr., Sect. C: Struct. Chem*. **2015**, *C71*, 9–18.

[S8] A. Datar, Y. G. Chung, L.-C. Lin, “Beyond the BET Analysis: The Surface Area Prediction of Nanoporous Materials Using a Machine Learning Method” *J. Phys. Chem. Lett*. **2020**, *11*, 5412−5417.

[S9] S. Lee, J. H. Lee, J. Kim, “User-friendly graphical user interface software for ideal adsorbed solution theory calculations” *Korean J. Chem. Eng.* **2018**, *35*, 214–221.

[S10] G. Kresse, J. Furthmüller, “Efficient iterative schemes for *ab initio* total-energy calculations using a plane-wave basis set” *Phys. Rev. B* **1996**, *54*, 11169–11186.

[S11] G. Kresse, J. Furthmüller, “Efficiency of ab-initio total energy calculations for metals and semiconductors using a plane-wave basis set” *Comput. Mater. Sci.* **1996**, *6*, 15–50.

[S12] G. Kresse, D. Joubert, “From ultrasoft pseudopotentials to the projector augmented-wave method” *Phys. Rev. B* **1999**, *59*, 1758–1775.

[S13] J. P. Perdew, K. Burke, M. Ernzerhof, “Generalized Gradient Approximation Made Simple” *Phys. Rev. Lett.* **1996**, *77*, 3865–3868.

[S14] R. W. Neuzil, “Aromatic hydrocarbon separation by adsorption” US 3,558,730, 1971.

[S15] Y. Yang, P. Bai, X. Guo, “Separation of Xylene Isomers: A Review of Recent Advances in Materials” *Ind. Eng. Chem. Res.* **2017**, *56*, 14725–14753.

[S16] H. van Koningsveld, A. J. van den Berg, J. C. Jansen, R. de Goede, “On a possible substitution of p-xylene by toluene in p-xylene crystals. The crystal structure of p-xylene, C8H10, at 180 K” *Acta Crystallogr., Sect. B: Struct. Sci.* **1986**, *42*, 491–497.

[S17] R. M. Ibberson, W. I. F. David, S. Parsons, M. Prager, K. Shankland, “The Crystal Structure of M-Xylene and p-Xylene, C_8_D_10_, at 4.5 K” *J. Mol. Struct.* **2000**, *524*, 121–128.

[S18] J. Marciniak, A. Katrusiak, “Direct and Inverse Relations between Temperature and Pressure Effects in Crystals: A Case Study on o-Xylene” *J. Phys. Chem. C* **2017**, *121*, 22303–22309.

[S19] D. S. Yufit, “The low-melting compounds 1,4-di­ethyl-, 1,2-diethyl- and ethyl­benzene” *Acta Crystallogr., Sect. C: Struct. Chem*. **2013**, *C69*, 273–276.

[S20] L. Li, L. Guo, D. Olson, S. Xian, Z. Zhang, Q. Yang, K. Wu, Y. Yang, Z. Bao, Q. Ren, J. Li, “Discrimination of xylene isomers in a stacked coordination polymer” *Science* **2022**, *377*, 335–339.

[S21] M. du Plessis, V. I. Nikolayenko, L. J. Barbour, “Record-Setting Selectivity for p-Xylene by an Intrinsically Porous Zero-Dimensional Metallocycle” *J. Am. Chem. Soc.* **2020**, *142*, 4529–4533.

[S22] L. Yu, J. Zhang, S. Ullah, J. Yao, H. Luo, J. Huang, Q. Xia, T. Thonhauser, J. Li, H. Wang, “Separating Xylene Isomers with a Calcium Metal-Organic Framework” *Angew. Chem., Int. Ed.* **2023**, *62*, e202310672.

[S23] X.-J. Xie, H. Zeng, Y.-L. Huang, Y. Wang, Q.-Y. Cao, W. Lu, D. Li, “Direct production of o-xylene from six-component BTEXs using a channel-pore interconnected metalorganic framework” *Chem* **2025**, *11*, 102339.

[S24] Z. Ye, X. Zhang, D. Liu, Y. Xu, C. Wang, K. Zheng, D. Zhou, C. He, J. Zhang, “A Gating Ultramicroporous Metal-Organic Framework Showing High Adsorption Selectivity, Capacity and Rate for Xylene Separation” *Sci. China-Chem.* **2022**, *65*, 1552–1558.

[S25] M. Rahmani, C. R. M. O. Matos, S.-Q. Wang, A. A. Bezrukov, A. C. Eaby, D. Sensharma, Y. Hjiej-Andaloussi, M. Vandichel, M. J. Zaworotko, “Highly Selective p-Xylene Separation from Mixtures of C8 Aromatics by a Nonporous Molecular Apohost” *J. Am. Chem. Soc.* **2023**, *145*, 27316–27324.

[S26] G.-Q. Guo, H. Chen, Y.-C. Long, “Separation of p-xylene from C8 aromatics on binder-free hydrophobic adsorbent of MFI zeolite. I. Studies on static equilibrium” *Microporous Mesoporous Mater.* **2000**, *39*, 149–161.

[S27] D. M. Polyukhov, A. S. Poryvaev, A. S. Sukhikh, S. A. Gromilov, M. V. Fedin, “Fine-Tuning Window Apertures in ZIF-8/67 Frameworks by Metal Ions and Temperature for High-Efficiency Molecular Sieving of Xylenes” *ACS Appl. Mater. Interfaces* **2021**, *13*, 40830–40836.

[S28] S. Laha, R. Haldar, N. Dwarkanath, S. Bonakala, A. Sharma, A. Hazra, S. Balasubramanian, T. K. Maji, “A Dynamic Chemical Clip in Supramolecular Framework for Sorting Alkylaromatic Isomers using Thermodynamic and Kinetic Preferences” *Angew. Chem., Int. Ed.* **2021**, *60*, 19921–19927.

[S29] Y. Lin, J. Zhang, H. Pandey, X. Dong, Q. Gong, H. Wang, L. Yu, K. Zhou, W. Yu, X. Huang, T. Thonhauser, Y. Han, J. Li, “Efficient Separation of Xylene Isomers by Using a Robust Calcium-Based Metal-Organic Framework through a Synergetic Thermodynamically and Kinetically Controlled Mechanism” *J. Mater. Chem. A* **2021**, *9*, 26202–26207.

[S30] N. Sun, S.-Q. Wang, R. Zou, W.-G. Cui, A. Zhang, T. Zhang, Q. Li, Z.-Z. Zhuang, Y.-H. Zhang, J. Xu, M. J. Zaworotko, X.-H. Bu, “Benchmark Selectivity p-Xylene Separation by a Non-Porous Molecular Solid through Liquid or Vapor Extraction” *Chem. Sci.* **2019**, *10*, 8850–8854.

[S31] K. B. Idrees, Z. Li, H. Xie, K. O. Kirlikovali, M. Kazem-Rostami, X. Wang, X. Wang, T.-Y. Tai, T. Islamoglu, J. F. Stoddart, R. Q. Snurr, O. K. Farha, “Separation of Aromatic Hydrocarbons in Porous Materials” *J. Am. Chem. Soc.* **2022**, *144*, 12212–12218.

[S32] M.-Y. Gao, S.-Q. Wang, A. A. Bezrukov, S. Darwish, B.-Q. Song, C. Deng, C. R. M. O. Matos, L. Liu, B. Tang, S. Dai, S. Yang, M. J. Zaworotko, “Switching Adsorbent Layered Material That Enables Stepwise Capture of C8 Aromatics Via Single-Crystal-to-Single-Crystal Transformations” *Chem. Mater.* **2023**, *35*, 10001–10008.

[S33] B. Moosa, L. O. Alimi, A. Shkurenko, A. Fakim, P. M. Bhatt, G. Zhang, M. Eddaoudi, N. M. Khashab, “A Polymorphic Azobenzene Cage for Energy-Efficient and Highly Selective p-Xylene Separation” *Angew. Chem., Int. Ed.* **2020**, *59*, 21367–21371.

[S34] M. Rasouli, N. Yaghobi, F. Allahgholipour, H. Atashi, “Para-Xylene Adsorption Separation Process Using Nano-Zeolite Ba-X” *Chem. Eng. Res. Des.* **2014**, *92*, 1192–1199.

[S35] M. Rasouli, N. Yaghobi, S. Z. Movassaghi Gilani, H. Atashi, M. Rasouli, “Influence of Monovalent Alkaline Metal Cations on Binder-Free Nano-Zeolite X in *para*-Xylene Separation” *Chin. J. Chem. Eng.* **2015**, *23*, 64–70.

[S36] F. Vermoortele, M. Maes, P. Z. Moghadam, M. J. Lennox, F. Ragon, M. Boulhout, S. Biswas, K. G. M. Laurier, I. Beurroies, R. Denoyel, M. Roeffaers, N. Stock, T. Düren, C. Serre, D. E. De Vos, “p-Xylene-Selective Metal–Organic Frameworks: A Case of Topology-Directed Selectivity” *J. Am. Chem. Soc.* **2011**, *133*, 18526–18529.

[S37] R. Lyndon, Y. Wang, I. M. Walton, Y. Ma, Y. Liu, Z. Yu, G. Zhu, S. Berens, Y.-S. Chen, S. G. Wang, S. Vasenkov, D. S. Sholl, K. S. Walton, S. H. Pang, R. P. Lively, “Unblocking a rigid purine MOF for kinetic separation of xylenes” *Chem. Commun.* **2022**, *58*, 12305–12308.

[S38] A. A. Sapianik, E. R. Dudko, K. A. Kovalenko, M. O. Barsukova, D. G. Samsonenko, D. N. Dybtsev, V. P. Fedin, “Metal-organic frameworks for highly selective separation of xylene isomers and single-crystal x-ray study of aromatic guest-host inclusion compounds” *ACS Appl. Mater. Interfaces* **2021**, *13*, 14768–14777.

[S39] X. Li, J. Wang, N. Bai, X. Zhang, X. Han, I. da Silva, C. G. Morris, S. Xu, D. M. Wilary, Y. Sun, Y. Cheng, C. A. Murray, C. C. Tang, M. D. Frogley, G. Cinque, T. Lowe, H. Zhang, A. J. Ramirez-Cuesta, K. M. Thomas, L. W. Bolton, S. Yang, M. Schröder, “Refinement of Pore Size at Sub-Angstrom Precision in Robust Metal–Organic Frameworks for Separation of Xylenes” *Nat. Commun.* **2020**, *11*, 4280.

# Author Contributions

S.L., A.S., and M.S.L. conceived the idea and designed the experiments. S.L., A.S., J.L. and H.C. synthesized and characterized the materials. J.H.L and S.K.M. conducted the calculations and simulations. All authors discussed the results, analyzed the data, and commented on the manuscript. M.S.L. supervised the overall study.
